# Landscape of surfaceome and endocytome in human glioma is divergent and depends on cellular spatial organization

**DOI:** 10.1073/pnas.2114456119

**Published:** 2022-02-25

**Authors:** Valeria Governa, Hugo Talbot, Kelin Gonçalves de Oliveira, Myriam Cerezo-Magaña, Anna Bång-Rudenstam, Maria C. Johansson, Ann-Sofie Månsson, Karin Forsberg-Nilsson, György Marko-Varga, Julio Enríquez Pérez, Anna Darabi, Johan Malmström, Johan Bengzon, Charlotte Welinder, Mattias Belting

**Affiliations:** ^a^Department of Clinical Sciences Lund, Section of Oncology, Lund University, Lund 221 85, Sweden;; ^b^Department of Immunology, Genetics, and Pathology, Science for Life Laboratory, Uppsala University, Uppsala 751 85, Sweden;; ^c^Clinical Protein Science and Imaging, Biomedical Centre, Department of Biomedical Engineering, Lund University, Lund 221 85, Sweden;; ^d^Chemical Genomics Global Research Lab, Department of Biotechnology, College of Life Science and Biotechnology, Yonsei University, Seoul 03722, Republic of Korea;; ^e^First Department of Surgery, Tokyo Medical University, Tokyo 160-8402, Japan;; ^f^Department of Clinical Sciences Lund, Section of Neurosurgery, Lund University, Lund 221 85, Sweden;; ^g^Department of Clinical Sciences Lund, Division of Infection Medicine, Lund University, Lund 221 85, Sweden;; ^h^Lund Stem Cell Center, Department of Clinical Sciences, Lund University, Lund 221 85, Sweden;; ^i^Department of Hematology, Oncology, and Radiophysics, Skåne University Hospital, Lund 221 85, Sweden

**Keywords:** glioma, immunotherapy, proteomics

## Abstract

Therapeutic strategies directed at the tumor surfaceome (TS), including checkpoint inhibitor blocking antibodies, antibody drug conjugates (ADCs), and chimeric antigen receptor T (CAR-T) cells, provide a new armament to fight cancer. However, a remaining bottleneck is the lack of strategies to comprehensively interrogate patient tumors for potential TS targets. Here, we have developed a platform (tumor surfaceome mapping [TS-MAP]) integrated with a newly curated TS classifier (SURFME) that allows profiling of primary 3D cultures and intact patient glioma tumors with preserved tissue architecture. Moreover, TS-MAP specifically identifies proteins capable of endocytosis as tractable targets for ADCs and other modalities requiring toxic payload internalization. In high-grade gliomas that remain among the most aggressive forms of cancer, we show that cellular spatial organization (2D vs. 3D) fundamentally transforms the surfaceome and endocytome (e.g., integrins, proteoglycans, semaphorins, and cancer stem cell markers) with general implications for target screening approaches, as exemplified by an ADC targeting EGFR. The TS-MAP platform was further applied to profile the surfaceome and endocytome landscape in a cohort of freshly resected gliomas. We found a highly diverse TS repertoire between patient tumors, not directly associated with grade and histology, which highlights the need for individualized approaches. Our data provide additional layers of understanding fundamental to the future development of immunotherapy strategies, as well as procedures for proteomics-based target identification and selection. The TS-MAP platform should be widely applicable in efforts aiming at a better understanding of how to harness the TS for personalized immunotherapy.

## Significance

Cancer immunotherapies, including checkpoint inhibitor blocking antibodies and antibody drug conjugates, currently revolutionize cancer treatment. However, a remaining challenge is the identification of tumor surfaceome (TS) targets for the design of more rational, individualized treatments. We have developed a procedure for unbiased mapping of TS targets in glioblastoma (GBM), i.e., the most common primary malignant brain tumor that remains among the most aggressive forms of cancer, and for which attempts to find effective treatments have failed so far. The present study provides additional layers of understanding fundamental to the future development of immunotherapy strategies, as well as procedures for proteomics-based target identification aimed at a better understanding of how to harness the TS for personalized immunotherapy.

Cell-surface proteins have a key role in drug development, and approximately two-thirds of approved human drugs target a cell-surface protein (1). Recently, tumor cell–surface proteins integrated with the plasma membrane (tumor surfaceome [TS]) have attracted considerable attention as targets for immunotherapies in cancer. Immune checkpoint-blocking antibodies (e.g., ipilimumab and nivolumab), antibody drug conjugates (ADCs, e.g., trastuzumab emtansin), radioimmunotherapy (RIT, e.g., ^90^Y ibritumomab tiuxetan), and chimeric antigen receptor T (CAR-T) cells are all directed at the TS and currently revolutionize cancer treatment (2–6). With the impressive development of creative methods for antibody and T cell engineering, a remaining challenge is the lack of strategies to comprehensively map potential TS target antigens for the design of more rational, individualized treatments (7). Although advancements in DNA and RNA sequencing provide high throughput data for prediction algorithms, e.g., personalized peptide vaccine trials (8, 9), the predicted proteome derived from these platforms is not necessarily expressed and available for targeting. Moreover, proteomics-based strategies involve analysis of the bulk from disintegrated tumor tissue, resulting in loss of spatial information and limited coverage of the less abundant and hydrophobic TS proteins (10, 11). Of particular relevance, ADC, RIT, and other intracellular drug delivery strategies rely on TS targets that functionally engage in endocytic internalization (12). Clearly, despite its great targeting potential in cancer immunotherapy, the TS remains an elusive treasure for further discovery.

Procedures for unbiased mapping of the TS and target identification should include specific labeling of the TS in freshly resected patient tumors with preserved tissue architecture. Enrichment of TS proteins and reduction of noise from intracellular proteins as well as abundant extracellular matrix collagens and glycoproteins would greatly improve downstream mass spectrometry analysis. Moreover, the approach should allow functional and dynamic profiling of TS internalization in an intact tissue environment. With the aim to address these challenges and to provide insight into the complexity of the TS, we have developed a versatile technology for TS mapping (TS-MAP). As proof of concept, we focused on primary brain tumors that remain among the most aggressive forms of cancer and for which attempts to conquer the most common variant, glioblastoma (GBM) (World Health Organization [WHO] grade IV) have failed so far (13). TS-MAP is compatible with spheroids from primary human stem cell–like GBM cultures, as well as mouse and patient brain tumors, and separately profiles surface resident and internalized TS proteins. Moreover, a TS classifier (SURFME) was curated for filtering and categorization of bona fide membrane proteins exposed to the extracellular space. We find significant differences in the TS between the 2D and 3D spheroid format, which underlines the importance of cellular spatial organization. In strong support of the need of individualized approaches, our findings suggest substantial intertumoral heterogeneity in the relative abundance of TS proteins in a cohort of freshly resected patient gliomas.

## Results

### TS-MAP in 3D and the SURFME Classifier.

**Fig. 1.**
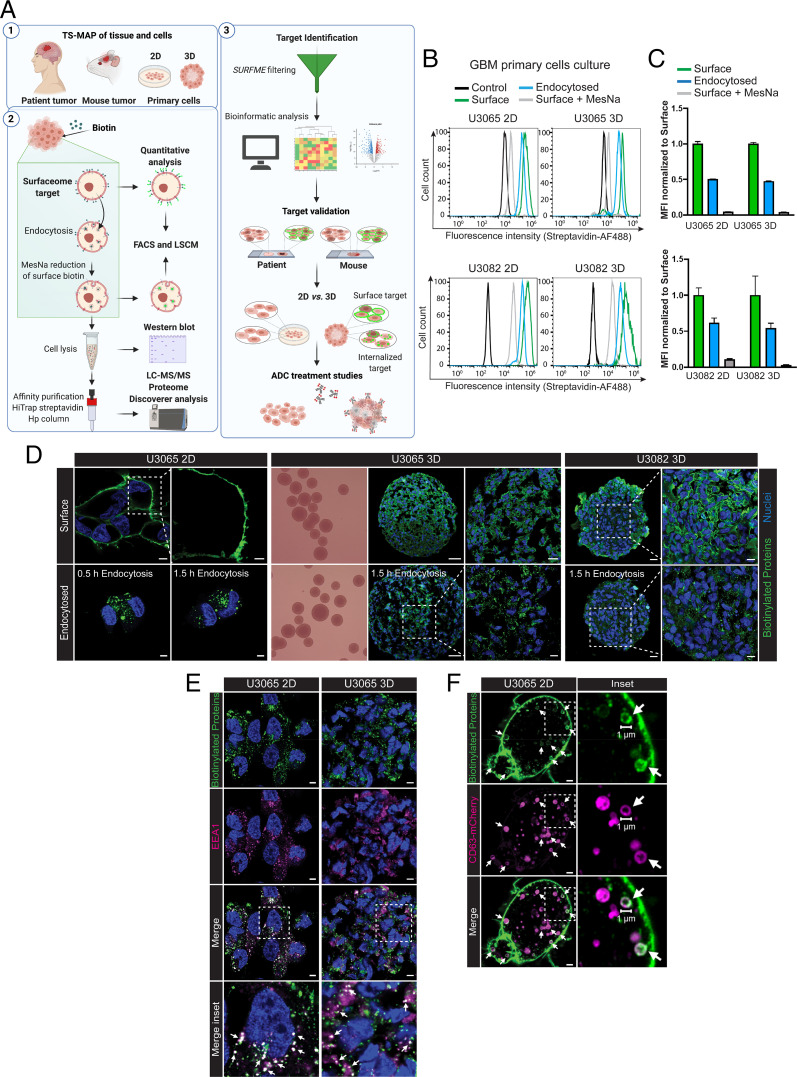
Tumor surfaceome mapping (TS-MAP) of glioma tumors. (*A*) Schematic outline of procedures for uncovering the surfaceome and endocytome in intact patient, mouse tumors, primary GBM 2D and 3D-spheroid cultures (*A*1). (*A*2) A workflow was established for reversible biotinylation, TS enrichment by high-affinity HPLC, and LC-MS/MS, integrated with high-resolution imaging using a GaAsP array-confocal detector (Airyscan) and FACS. (*A*3) An in-house TS classifier (SURFME) was curated for filtering and categorization of bona fide membrane proteins, identifying potential target candidates for further validation by immunofluorescence analyses of matched tumor sections, and pilot ADC treatment experiments in vitro. (*B*) Quantification of surfaceome and endocytome in primary GBM cell cultures grown in 2D and 3D. Representative histograms from FACS analysis of nonbiotinylated (control), total surface biotinylation (surface), residual cell-surface signal following MesNa treatment (surface + MesNa), and endocytosed surface proteins (internalized) in U3065 (*Upper*) and U3082 (*Lower*) GBM cells, grown in 2D or 3D, as indicated. (*C*) Quantitative analysis of the experiment presented in *B* with endocytosed proteins expressed as a fraction of the total surfaceome protein abundance. Data are presented as mean ± SD from three independent experiments each performed in triplicates. MFI, median fluorescence intensity. (*D*) High-resolution Airyscan imaging of surface and endocytosed biotinylated proteins (green) in GBM cells grown in 2D or 3D (shown in representative brightfield images), as indicated. (Scale bars, 5 μm [U3065 2D], 2 μm [U3065 2D *Inset*], 50 μm [U3065 and U3082 3D, *Left*], and 20 μm [U3065 and U3082 3D, *Left*].) (*E*) Airyscan imaging of endocytosed biotinylated proteins (green) and the early endosome marker EEA1 (magenta) in GBM cells grown in 2D or 3D, as indicated, after 1.5 h of endocytosis. (Scale bars, 5 μm, and 2 μm for *Insets*.) (*F*) Airyscan imaging for visualization of endocytosed surface proteins in the membrane of endolysosomal vesicles, as indicated by CD63-mCherry (magenta). Images were captured 45 min after initiation of endocytosis (see also Movies S1 and S2). (Scale bars, 2 μm.) (*D*–*F*) Shown are representative images from at least three independent experiments. White squares indicate zoomed areas shown in *Insets*. White arrows indicate colocalization.

We set out to develop a platform for tumor surfaceome and endocytome mapping (TS-MAP) that was compatible with human primary stem cell like GBM cells grown in 2D or as 3D-spheroid cultures. As outlined in Fig. 1*A*, the TS-MAP strategy was subsequently adopted for mouse GBM in vivo models and patient GBM tumors. We initially used primary GBM cells (U3065 and U3082) from the human GBM cell culture (human glioblastoma cell culture [HGCC]) resource (14) to perform comparative quantification of the global surfaceome and the internalized surfaceome fraction in 2D vs. 3D cultures (Fig. 1*B*). We could show that surface biotinylation efficiency in intact 3D spheroids on a per-cell basis was comparable to 2D cultures, and that ∼50% of the surfaceome was internalized in both culture formats over a period of 90 min (Fig. 1*C*). The biotinylation reagent used (EZ-Link Sulfo-*N*-hydroxysuccinimide-SS-biotin) specifically conjugates extracellularly exposed primary amines or amino termini of polypeptides of surface proteins. Its size and negative charge, provided by the sulfonated group, prevents direct cell membrane interaction, e.g., with neutral lipids and unspecific permeation. This was supported by imaging studies, showing comprehensive cell-surface labeling and distinct vesicular structures from internalized proteins in 2D cultures as well as in spheroids (Fig. 1*D* and *SI Appendix*, Fig. S1*A*). Importantly, treatment with MesNa, a membrane-impermeable reducing agent, efficiently abrogated residual cell-surface biotinylation in 2D and 3D cultures, as quantified by fluorescence-activated cell sorting (FACS) (Fig. 1*B*) and visualized by imaging and Western blotting (*SI Appendix*, Fig. S1 *B* and *C*). We could conclude that the internalized protein signal was not derived from any remaining surface-biotinylated proteins. To corroborate that the occurrence of intracellular biotinylation signal was associated with an endocytic process, GBM cell cultures were costained for biotin and the early endosome antigen 1 (EEA1), showing a clear colocalization in both 2D and 3D models (Fig. 1*E*). We employed GBM cells expressing the endolysosomal marker CD63 fused with mCherry and live high-resolution confocal microscopy to track translocation of biotinylated surface proteins toward intracellular, CD63-positive vesicles (Fig. 1*F* and Movie S1). Moreover, using fluorescently labeled ligands of macropinocytosis-dependent and membrane raft-mediated endocytosis (dextran and cholera toxin subunit B, respectively), we confirmed that intracellular biotinylation is the result of endocytic internalization of the surfaceome (*SI Appendix*, Fig. S1*D*).

**Fig. 2.**
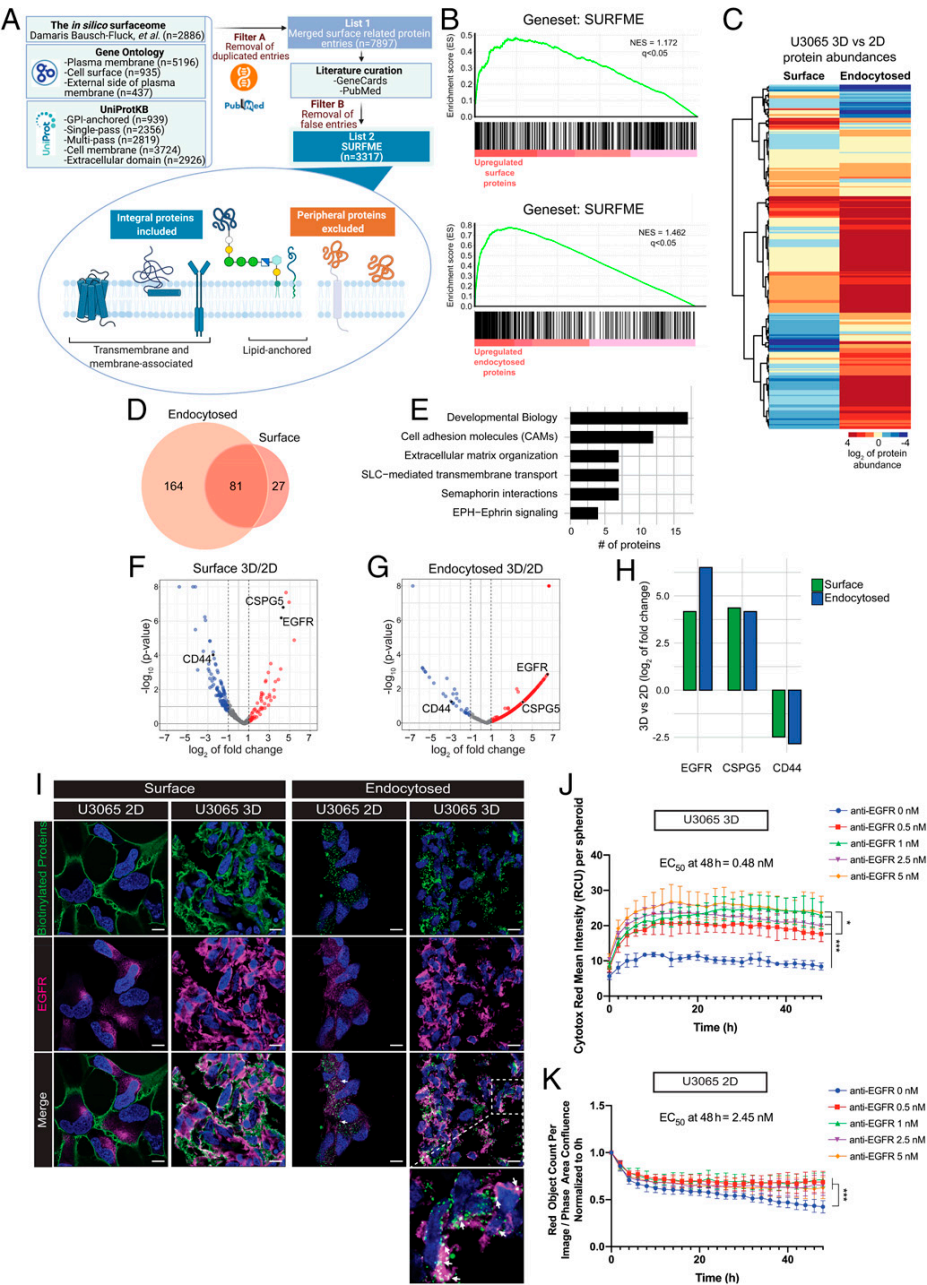
Influence of cellular spatial organization on glioma cell surfaceome and endocytome. (*A*) Construction of the SURFME classifier. To allow filtering of LC-MS/MS data for putative TS targets, a machine learning–based predictor that excludes membrane proteins integrated on the cytoplasmic side (1) was merged with GO terms, as indicated, as well as categorization into single and multipass transmembrane proteins and glycosyl-phosphatidyl-insositol (GPI)-anchored proteins according to reviewed (Swiss-Prot), and manually annotated hits in UniProt. Duplicated hits were removed (filter A), followed by in-depth interrogation of GO, GeneCards, and PubMed, excluding additional hits (filter B) not fulfilling the criteria (see *Lower* panel). This generated a final SURFME classifier encompassing 3,317 proteins. (*B*) Validation of TS-MAP biotinylation and HPLC strategy. GSEA shows significant enrichment of SURFME identities, identified in GBM cells by LC-MS/MS, in “surface” (*Upper*) and “endocytosed” (*Lower*) biotinylated samples as compared with nonbiotinylated control sample. (*C*) Surfaceome protein abundance heatmap demonstrates divergent surfaceome and endocytome in 3D vs. 2D GBM cell cultures. (*D*) Venn diagram of the unique and overlapping protein identities of surfaceome proteins up-regulated (≥0.5 log_2_ FC) in surface and/or endocytosed samples from 3D cultures compared to 2D. (*E*) Overrepresented pathways for SURFME proteins up-regulated in 3D vs. 2D that relate to tissue organization. (*F*) Volcano plot displays magnitude of SURFME protein changes (log_2_ fold) vs. statistical significance (*P* value) at the surface of 3D compared with 2D cultures. (*G*) Same as in *F* for endocytome. (*H*) Relative abundance of the indicated proteins at the surface and endocytosed in 3D vs. 2D cultures. (*I*) Immunofluorescence validation: 2D and 3D cultures, as indicated, were surface biotinylated and allowed to endocytose (endocytosed) or not (surface). The 2D cells and spheroid cryosections were then stained with streptavidin-AF488 (green) and anti-EGFR antibody (magenta). Shown are representative images from at least three independent experiments. White squares indicate zoomed areas shown in *Insets*. White arrows indicate colocalization. (Scale bars, 10 μm.) (*J* and *K*) Treatment with increasing concentration of anti-EGFR ADC was initiated at *t* = 0 and cytotox mean fluorescence intensity in spheroids (*J*) or red object count normalized to confluence in 2D (*K*) were measured every 2 h. Data are presented as the mean ± SD from four independent replicates.

We next built an in-house surfaceome identification (ID) classifier (Fig. 2*A*) by concatenating single and multipass transmembrane proteins, as well as glycosyl-phosphatidyl-insositol (GPI)-anchored proteins from reviewed (Swiss-Prot) and manually annotated hits using the terms “cell membrane” and “extracellular domain” in UniProt (https://www.uniprot.org/). Additional hits were obtained from gene ontology (GO; http://geneontology.org/) terms “plasma membrane,” “cell surface,” and “external side of plasma membrane.” Retrieved protein IDs were then matched against the SURFY predictor, i.e., a machine learning–based predictor that excludes membrane proteins integrated on the cytoplasmic side lacking an extracellularly exposed motif (http://wlab.ethz.ch/surfaceome) (1). As the reported accuracy of SURFY was 93.5%, the entire SURFY profile (*n* = 2,886 proteins) was further considered as true hits. Importantly, we identified additional proteins (*n* = 431), not included in SURFY, by in-depth interrogation of UniProt, GeneCards, GO, and PubMed. We finally curated a total catalog of 3,317 proteins, hereafter referred to as SURFME (available in Dataset S1), which corresponds to ∼16% of the predicted human proteome. SURFME included GPI-anchored (*n* = 140), α-helical single-pass transmembrane (*n* = 1,316), and multipass transmembrane (*n* = 1,750) proteins as well as a small subset (*n* = 116) of proteins (∼3.5% of SURFME IDs) that did not fit into the major categories (*SI Appendix*, Fig. S2*A*).

We next applied the SURFME as a “geneset” within a gene set enrichment analysis (GSEA). In support of the TS-MAP strategy, “surfaceome” and “endocytome” proteins identified in GBM cells displayed a strong enrichment for SURFME when compared to control, nonbiotinylated samples (Fig. 2*B*). Moreover, SURFME filtering revealed a potential overrepresentation of single-pass transmembrane proteins among experimentally identified surfaceome proteins (∼62% of total identities) vs. the theoretical SURFME set (39.5% of total identities) (*SI Appendix*, Fig. S2*B*). Together, we conclude that TS-MAP permits specific enrichment and detection of internalized, low abundant membrane proteins by reducing the contribution of abundant cytosolic and nuclear proteins.

### Tumor Cell Spatial Organization Transforms the Surfaceome and Endocytome in Glioma.

Establishment of the TS-MAP procedure in a 3D-spheroid model allowed us to investigate how the global surfaceome and endocytome is dictated by tumor cell architecture. We found a remarkable divergence in the relative abundance of surfaceome and endocytome proteins in 3D as compared with 2D models. Interestingly, primary glioma cell organization into spheroids was associated with increased internalization of a dominating pool of cell-surface proteins, as shown by differentially expressed protein (DEP) analysis (Fig. 2*C*). Among SURFME proteins up-regulated in 3D vs. 2D (fold-change [FC] cutoff = 0.5 log_2_), 27 were exclusively up-regulated at the surface, 81 were up-regulated at the surface and had increased internalization, and 164 were more endocytosed (Fig. 2*D* and Dataset S2). In general, up-regulated proteins, both surface resident and endocytosed, were mostly classified as single-pass transmembranes (*SI Appendix*, Fig. S2*C*). We found induced expression of several members of the integrin (e.g., ITGAV, ITGB5, ITGA7, ITGB4, and ITGA6), proteoglycan (e.g., BCAN, CSPG5, and GPC1), and semaphorin (SEMA5A and SEMA6D) families in the surfaceome and/or endocytome of 3D as compared with 2D cultures. Accordingly, among overrepresented pathways in spheroids, we identified, e.g., “cell adhesion molecules,” “developmental biology,” “extracellular matrix organization,” and “semaphorin interactions,” which reflect cell–cell and cell–matrix interactions expected to be involved in 3D cellular organization (Fig. 2*E*). Also, consistent with spheroid formation, we found induction of hypoxia- and acidosis-regulated proteins (SLC16A1/MCT1 and SLC4A4). Interestingly, several proteins associated with cancer stem cells, including in glioma [CD166 (15), F2R/PAR1 (16), EDNRB (17), CX3CL1 (18), ITGA6 (19), and ITGA7 (20)] displayed induced surfaceome or endocytome expression in 3D vs. 2D cultures.

We next selected some of the identified candidate hits, according to 3D vs. 2D differential expression of ≥0.5 log_2_ FC or ≤ −0.5 log_2_ FC in surface and endocytosed datasets, for immunofluorescence validation (Fig. 2 *F*–*H*). In concordance with the TS-MAP data, we found EGFR overexpression in 3D as compared with 2D cultures (Fig. 2 *I*, *Left*), and could observe intracellular colocalization of EGFR with endocytosed proteins in spheroids, while this appeared relatively scarce in 2D cultures (Fig. 2 *I*, *Right*). Similar results were obtained with CSPG5 (*SI Appendix*, Fig. S2*D*), i.e., another candidate from TS-MAP analysis (Fig. 2 *F*–*H*). We could further corroborate the down-regulation of CD44 in 3D vs. 2D cultures (*SI Appendix*, Fig. S2*E*).

A relevant ADC target should preferentially be highly expressed at the cell surface but also efficiently endocytosed to deliver the cytotoxic payload. To understand whether the increased surface availability and constitutive endocytosis of EGFR in spheroids translated into an increased ADC effect, we next treated 2D and 3D glioma cultures with an anti-EGFR antibody precomplexed with a secondary antibody-monomethyl auristatin F toxin conjugate. We employed an Incucyte imaging system to dynamically follow ADC-induced cytotoxicity in 2D and 3D models over time (*SI Appendix*, Fig. S2 *F* and *G*). In support of increased EGFR-mediated ADC delivery in spheroids, we observed an estimated half maximal effective concentration (EC_50_) of 0.48 and 2.45 nM in the 3D and 2D cultures, respectively (Fig. 2 *J* and *K*).

Together, our results provide a complex understanding of how the surfaceome and endocytome are transformed during spheroid formation, a process generally linked to cancer cell stemness (21). Moreover, validation stainings and ADC treatment studies establish TS-MAP as a high throughput platform for exploration of potential target antigens in vitro.

### TS-MAP Reveals Divergent Surfaceome Landscapes in Patient Gliomas.

Although TS-MAP compatibility with intact spheroids is a significant advancement over conventional 2D models, in vitro models fail to fully reflect the complexity of the in vivo situation. To expand on the utility of the TS-MAP approach in vivo, we initially employed a syngeneic mouse GBM model (GL261) known to recapitulate many of the histological features of human GBM (22). GL261 cells were orthotopically injected into the right hemisphere, and following 33 d of incubation, freshly resected tumors as well as nontumor brain tissue from the contralateral hemisphere were processed through the TS-MAP and SURFME platform (*SI Appendix*, Fig. S3*A*). We found efficient and comprehensive surface biotinylation of intact tumor tissue sections (*SI Appendix*, Fig. S3*B*), as well as in single-cell suspensions (SCSs) from disintegrated tumor and normal mouse brain (*SI Appendix*, Fig. S3 *C* and *D*). SURFME filtering and DEP analysis of liquid chromatography with tandem mass spectrometry (LC-MS/MS) data consistently showed an expected divergence in surface protein abundances between tumor and normal brain (*SI Appendix*, Fig. S3*E* and Dataset S3). Out of 346 identified SURFME proteins, 117 were up-regulated in tumor tissue when compared to normal brain, comprising mostly single-pass transmembrane proteins (*SI Appendix*, Fig. S3*F*). Similar pathways were overrepresented in tumor vs. normal brain (*SI Appendix*, Fig. S3*G*) as found in 3D vs. 2D (Fig. 2*E*), and included additional tumoral processes, such as “PI3K-Akt signaling pathway” and “integrin cell surface interactions.” Interestingly, GPNMB, the most up-regulated SURFME protein in tumor vs. normal brain (*SI Appendix*, Fig. S3*H*) was previously identified as a tumor-associated surface antigen in human GBM (23), and a targeting ADC (glembatumumab vedotin) has entered clinical trials with several tumor types (24). Conversely, the most down-regulated protein, HCN1 (*SI Appendix*, Fig. S3*H*), is known as a pacemaker channel widely expressed in the mouse and human brain (25). We conclude that TS-MAP combined with SURFME filtering is compatible with ex vivo surfaceome profiling of intact GBM tumors.

**Fig. 3.**
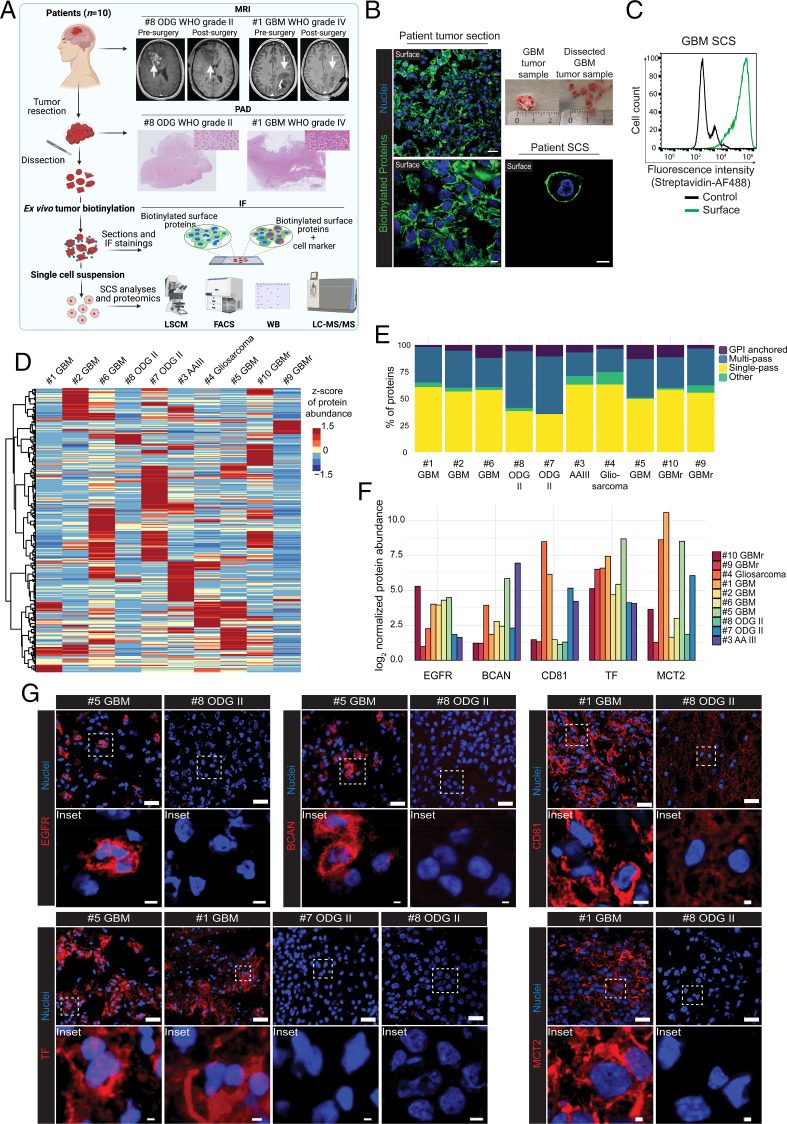
TS-MAP uncovers a divergent surfaceome landscape in patient gliomas. (*A*) TS-MAP was applied in a mixed cohort (*n* = 10) of patient gliomas. Shown are MRI (presurgery and postsurgery within 48 h) and histology for pathological-anatomical diagnostics (PAD) according to clinical routine, here exemplified by patient #8ODG with a low-grade ODG and #1GBM representing a high-grade GBM. Freshly resected tumors were biotinylated ex vivo for downstream analyses, as indicated. (*B*) Confocal microscopy shows biotinylation (green) of intact patient tumor. Higher magnification (*Bottom*) indicates surface labeling, which is further supported by Airyscan imaging of disintegrated SCS (*Bottom Right*), showing specific plasma membrane labeling. *Top Right* shows example of fresh tumor dissection into pieces of 0.3 to 0.5 cm in diameter prior to biotinylation (ruler scale in centimeters). (Scale bars, 20 μm [*Top Left*], 5 μm [*Bottom Left* and *Right*].) (*C*) FACS quantification of streptavidin-AF488 association with nonbiotinylated (control) and biotinylated (surface) in a representative GBM patient tissue SCS. (*D*) TS-MAP was applied in a pilot cohort of 10 freshly resected patient tumors, including low-grade ODG (WHO grade II), high-grade anaplastic astrocytoma (AA, WHO grade III), primary glioblastoma (GBM, WHO grade IV), recurrent GBM (GBMr), and gliosarcoma (WHO grade IV). Heatmap of SURFME protein abundance demonstrates divergent expression profile among patient tumors. (*E*) Categories of SURFME proteins relatively up-regulated in the respective patient tumor. (*F*) Quantification of normalized, relative abundance (log_2_ fold) of selected SURFME proteins expressed in different patient tumors. Normalization was done based on the sample with the lowest abundance for each SURFME protein. EGFR, BCAN, and TF are normalized to #8ODG II abundances, CD81 is normalized to #2GBM abundance, and MCT2 to #3AA III. (*G*) Validation of LC-MS/MS data by immunofluorescence of selected SURFME proteins in matched patient tumor sections. Shown are representative images from at least three independent experiments. White squares indicate zoomed areas shown in *Insets*. (Scale bars, 20 μm, and 2 or 5 μm for narrow and wide bars, respectively, in *Insets*.)

We next adopted the TS-MAP platform to interrogate the surfaceome landscape in a pilot cohort (*n* = 10) of patient gliomas covering different WHO grades and histologies (Fig. 3*A*; patient characteristics are presented in *SI Appendix*, Table S1). Following routine MRI examination, and in parallel with pathological-anatomical diagnosis (PAD), fresh tumor specimens were dissected into 0.3- to 0.5-cm pieces (Fig. 3*B*) and further processed according to the TS-MAP workflow. We could verify comprehensive and distinct cell-surface labeling of intact patient tumors by immunofluorescence imaging of tissue sections and FACS analysis of tumor SCSs (Fig. 3 *B* and *C*). We found a strikingly heterogeneous SURFME protein expression between patient tumors, and DEP analysis depicted a unique pattern of overexpressed proteins apparently unrelated to histology or grade (Fig. 3*D*). Hotspots of highly expressed SURFME proteins diverged according to subcategory, with a potential enrichment of multipass transmembrane proteins in oligodendrogliomas (ODGs) as compared with astrocytomas (Fig. 3*E*). We selected EGFR, BCAN, CD81, TF, and MCT2 for further studies based on their divergent, normalized relative abundances between high- and low-grade tumors according to TS-MAP/SURFME data (Fig. 3*F*). Another criterion for our candidate selection was that EGFR and TF are currently explored as ADC targets in clinical trials, and previous studies pointed at their potential as treatment targets in cancer (26–31). We next performed immunofluorescence stainings of available matched patient tumors for EGFR, TF, BCAN, CD81, and MCT2. In accordance with the TS-MAP profiling data, we found a clear overexpression of EGFR and TF in GBM as compared with ODG (Fig. 3*G*). Moreover, BCAN was highly expressed in patient tissue #5GBM as compared to #8ODG, and both MCT2 and CD81 were strongly expressed in #1GBM vs. #8ODG patient tissue (Fig. 3*G*). Importantly, as part of the validation, the selected proteins were preferentially expressed at the cell surface in tumor sections.

Solid tumors, including gliomas, are characterized by the infiltration of a variety of stromal and immune cells that contribute to the malignant process (32). In particular, reprogrammed vascular cells and immunosuppressive tumor-associated macrophages (TAMs) are prevalent in GBM, and their eradication has emerged as a potential treatment strategy (33, 34). To understand whether the TS-MAP approach included these GBM-microenvironment subsets, we performed immunofluorescence costaining for cell-surface biotinylation and specific markers of immune cells (CD45^+^), TAMs (CD68^+^), endothelial cells (CD31^+^), and EGFR^+^ cells (*SI Appendix*, Fig. S4). We consistently found a clear biotinylation labeling of these cell entities in tissue sections (*SI Appendix*, Fig. S4*A*), and confocal microscopy of tumor SCSs could confirm distinct surface biotinylation of CD45^+^, CD68^+^, CD31^+^, and EGFR^+^ tumor cells (*SI Appendix*, Fig. S4*B*). However, not only tumor resident but also systemic immune cells are of interest in cancer. Therefore, we isolated peripheral blood mononuclear cells (PBMCs; CD45^+^) from GBM patient blood for surface biotinylation and further enrichment of CD14^+^ monocytes. FACS analysis revealed comprehensive labeling of PBMCs and monocytes (90 to 95% of total) (*SI Appendix*, Fig. S4*C*), and specific cell-surface labeling was visualized by confocal microscopy (*SI Appendix*, Fig. S4*D*). Together, we conclude that patient tumors exhibit a remarkable TS heterogeneity that apparently is not associated with grade and histology, which reinforces the need for techniques allowing individualized target identification. TS-MAP may fulfill this need by providing a platform for direct surfaceome profiling of tumors and, in parallel, specific subsets of peripheral cells that could be used for target selection, but also improved diagnosis and monitoring.

### Decrypting the Global Endocytome in Patient Glioma.

**Fig. 4.**
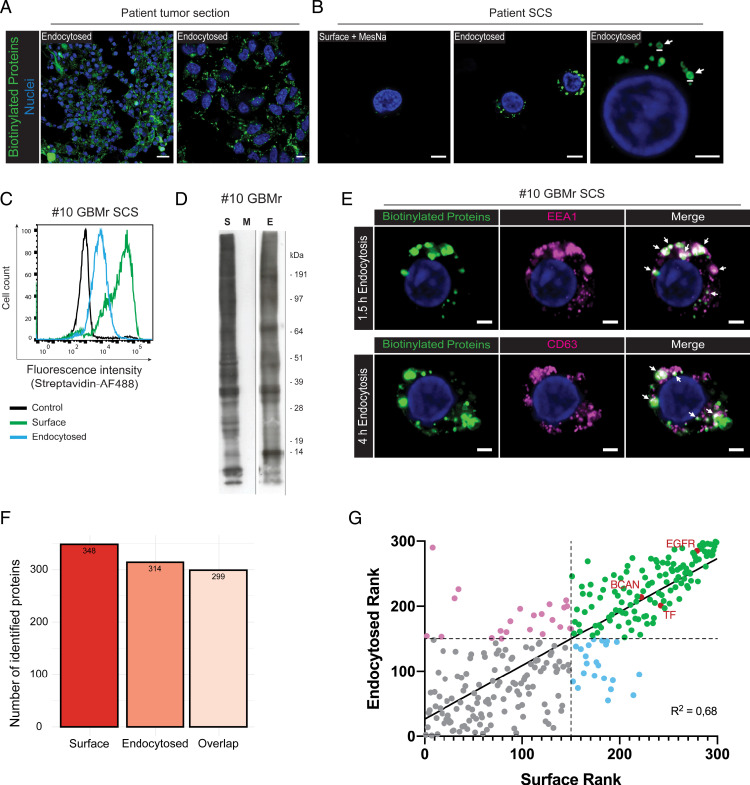
TS-MAP reveals a subset of highly endocytosed surface proteins in patient glioma. (*A*) Freshly resected, intact patient tumor specimens were cell-surface biotinylated and then put under endocytosis permissive conditions ex vivo. Cryosections were stained for internalized, biotinylated proteins with streptavidin-AF488 (green). Shown are representative images from two independent experiments at lower (*Left*) and higher (*Right*) magnification. (Scale bars, 20 μm [*Left*], 5 μm [*Right*].) (*B*) Tumor specimens were biotinylated as in *A* and then disintegrated into SCS for Airyscan imaging analysis. MesNa treatment completely abolished cell-surface biotinylation of tumors not allowed to perform endocytosis (surface + MesNa), while biotinylated proteins were confined to intracellular structures following internalization, as shown at lower (*Middle*) and higher (*Right*) magnification. White arrows indicate endocytic vesicles of 0.5 to 1 μm in diameter. (Scale bars, 5 μm.) (*C*) FACS quantification of streptavidin-AF488 staining of nonbiotinylated (control) and biotinylated (surface and endocytosed) proteins in SCS from #10GBMr. (*D*) Western blotting of biotinylated proteins from similar experiment as in *D* shows efficient surfaceome (S) and endocytome (E) labeling, and removal by MesNa (M). (*E*) Similar experiment as in *B* shows clear colocalization (white arrows) of internalized proteins (green) with the early endosome marker EEA1 and the endolysosomal marker CD63 (magenta). (Scale bars, 2 μm.) (*F*) TS-MAP was applied to the #10GBMr patient sample and LC-MS/MS proteomic data of surface and endocytosed protein identities was filtered with SURFME. Bar plot shows the number of SURFME proteins that were identified, respectively, in surface and endocytosed samples and their overlap. (*G*) SURFME proteins identified in the surfaceome as well as the endocytome of #10GBMr were ranked according to their abundance in the respective location (lowest = 1, highest = 299), and divided into four categories. *Top Right*, proteins with relatively high surface abundance and efficient endocytic capacity.

We finally wanted to explore the possibility of maintaining surfaceome-labeled patient glioma specimens under endocytosis permissive conditions to profile the endocytome of intact tumor tissue. After extensive method optimization, we chose 1.5 h as a good time point of constitutive endocytosis, as this yielded a clearly visible, intracellular signal in tumor sections (Fig. 4*A*). Importantly, we could confirm efficient internalization into vesicular structures as well as eradication of remaining surface biotinylation by MesNa, using high-resolution imaging (Fig. 4*B*), FACS (Fig. 4*C*), and Western blotting (Fig. 4*D*). As further evidence of endocytic internalization in this setting, the intracellular biotinylation signal showed colocalization and time-dependent transition from early endosomes (EEA1^+^) to late endolysosomes (CD63^+^) (Fig. 4*E*). From these data, we were encouraged to profile the surfaceome and endocytome from a patient with recurrent GBM (#10GBMr). LC-MS/MS analysis and SURFME filtering identified 348 surface proteins, out of which 299 were also endocytosed (Fig. 4*F*). We found an additional 49 proteins expressed exclusively at the surface and 15 confined to the endocytosed fraction (Fig. 4*F*). We argued that proteins that are strongly expressed at the surface and efficiently endocytosed represent targets with a potential application for ADC or RIT. By ranking identified SURFME proteins according to their relative abundance in the surfaceome and endocytome, we selected identities with high-abundance ranking in both fractions (Fig. 4 *G*, *Top Right* quadrant) for analysis of matched tissue sections (see *SI Appendix*, Table S2 for a detailed abundance ranking of surfaceome and endocytome proteins). Using high-resolution confocal microscopy, we could observe a strong surface and intracellular signal of EGFR, BCAN, and TF in #10GBMr, thus corroborating the LC-MS/MS data (*SI Appendix*, Fig. S5). Altogether, these results provide proof of concept of the TS-MAP platform as a versatile tool to profile the highly diverse tumor surfaceome and functional endocytome in patient tumors.

## Discussion

With the advent of cancer immunotherapies, strategies for surfaceome profiling have come into great focus (35). Here, we have established the TS-MAP technology, which represents an important advancement over current RNA-based and 2D-surfaceome approaches by providing additional layers of understanding related to cellular spatial organization (2D vs. 3D) and protein localization (surface resident vs. endocytosed). The TS-MAP workflow was designed to allow surfaceome and endocytome mapping in fresh patient tumors and adds biological parameters fundamental to immunotherapy target selection for further drug development.

As proof of concept, we have applied the TS-MAP technology to map surface resident and endocytosed proteins in primary GBM cells grown in 2D or as spheroids. Our findings reveal substantial remodeling of the surfaceome and endocytome landscapes during spatial reorganization of GBM cells. Specifically, we find that several integrins, proteoglycans, as well as proteins previously implicated in glioma cancer stem cell biology and treatment resistance are induced in 3D cultures. As part of the validation, we show that EGFR, one of the SURFME proteins induced in the 3D surfaceome as well as endocytome, could be targeted by an ADC construct specifically in the 3D setting.

Although versatile, the purification of biotinylated surface proteins remains prone to contaminations from associated protein complexes and unspecific protein interactions (36, 37). This has generated numerous surfaceome catalogs for LC-MS/MS data filtering based on bioinformatic annotations from RNA data (1, 38) or combined with experimental proteomics data from 2D cell cultures (1, 39–42). In the present study, we applied stringent washing steps prior to reductive elution of biotinylated proteins from the streptavidin resin to minimize contamination from unspecific interactions. In addition, we constructed an updated curation of transmembrane or GPI-linked proteins, SURFME, that accurately selects for membrane proteins exposing an extracellular domain.

We further developed the TS-MAP approach for unbiased profiling of mouse and patient gliomas with preserved tissue architecture. TS-MAP and SURFME filtering was applied on a pilot cohort of patient gliomas, revealing substantial intertumoral heterogeneity. It is conceivable that our findings partly reflect the oncogenetic multiclonality and transcriptional diversity of highly malignant tumors, including GBM (13). This warrants future studies that elucidate how the oncogenetic profile and spatial organization synergize to shape the full complexity of the GBM surfaceome with direct implications for cancer stem cell and infiltrative cellular behavior, i.e., major challenges of this incurable disease. Interestingly, Leung et al. recently applied techniques for comprehensive mapping of *N*-glycosylated cell-surface proteins to reveal how key oncogenes can remodel the surfaceome in breast epithelial cells (40). However, at this stage, we can only speculate on the relative contribution of the malignant cell and nonmalignant cell compartments to the intertumoral surfaceome heterogeneity. Likely, it is the combined ecosystem of interactions between these compartments that will shape the diverse surfaceome. Future studies should explore the prospect of using the surfaceome profile as a predictor of clinical outcome. Again, it is conceivable that the combined surfaceome of malignant and nonmalignant cells will contribute prognostic information and to the prediction of treatment response. The TS-MAP platform can also be generally applied to elucidate global effects of conventional oncological treatment (radiochemotherapy) by comparing primary and recurrent patient tumors. This should provide useful insights into the design of adjuvant immunotherapies specifically targeted at resistance mechanisms.

Moreover, TS-MAP could be expanded for the purpose of noninvasive brain tumor diagnosis that remains a challenge of high clinical relevance (43). Extracellular vesicles (EVs) have received considerable attention as potential biomarkers as they represent “a miniature of its cell of origin” (44). EVs are derived directly from the plasma membrane (microvesicles) or from the endolysosomal system (exosomes), and thus carry a large repertoire of the tumor surfaceome and endocytome into circulation (45). Recently, we identified the surface protein syndecan 1 (SDC1) in tumor-derived EVs, and plasma levels of EV-SDC1 could discriminate between GBM and low-grade glioma (46). Hence, state-of-the-art methods for plasma EV isolation combined with the TS-MAP approach may provide unique opportunities for dynamic monitoring and possibly therapeutic stratification.

Clearly, many challenges lay ahead, and one limitation of our study is surfaceome/endocytome mapping on the tumor bulk, including malignant and nonmalignant tumor cells. While providing a comprehensive view of the entire tumor microenvironment, downstream analysis by, e.g., immunofluorescence is required to verify target protein expression on the cells of interest. However, we have demonstrated that TS-MAP is compatible with tissue disintegration into SCSs with preserved biotin labeling, which together with FACS sorting and ultrasensitive mass spectrometry approaches may even allow single-cell surfaceome profiling of patient tumors. The present work should make an important addition to ongoing efforts in spatial transcriptomics, as the TS-MAP platform may identify cell–cell interactions at the surface protein level and in a spatially restricted manner. Moreover, our studies show efficient biotin labeling of the entire PBMC population and successful magnetic bead sorting of biotinylated subpopulations (CD14^+^). The TS-MAP approach could be integrated with downstream sorting of malignant cells and tumor infiltrating immune cell populations employing positive and negative selection based on immunoabsorption and FACS sorting. This motivates further development into dynamic and comparative surfaceome profiling of tumor-associated immune cells, including macrophages, neutrophils, T cells, and their peripheral counterparts.

In the case of brain tumors, a specific challenge is imposed by the blood–brain barrier that constitutes a barrier to macromolecular drugs. However, several ADC therapies have been developed for brain tumors (47), and clinical trials with Depatux-M targeted at EGFR generated some long-term responses in patients with recurrent, EGFR-amplified GBM (26). The TS-MAP approach, together with genomic profiling data, point at the need of sequential and more rational multitargeting strategies with, e.g., bispecific, or even trispecific antibodies that are still in preclinical development (48). Such strategies may allow increased tumor cell specificity as well as the recruitment of cytotoxic T cells (tumor infiltrating lymphocytes [TILs]) to the tumor site.

To conclude, we have established the TS-MAP platform and SURFME classifier that should be widely applicable to a variety of solid tumors in efforts aiming to harness the surfaceome for personalized immunotherapy. Our findings in GBM provide unique insights into the complexity of the TS and highlight the need of TS-MAP and similar techniques for separate profiling of the surfaceome and endocytome in individual tumors.

## Methods

Detailed descriptions of the mouse GBM model, Western blotting, ligand uptake, biotinylation of PBMCs and CD14^+^ magnetic cell separation sorting, Incucyte cytotox assay, and GSEA and pathway analysis of LC-MS/MS data are listed in *SI Appendix*.

### Patient-Derived Primary GBM Cells.

U3065-MG and U3082-MG cells were obtained from the HGCC biobank, Uppsala University, Uppsala, Sweden (14). Cells were seeded on dishes precoated with 10 μg/mL poly-L-ornithine (Sigma-Aldrich, P3655) and 10 μg/mL laminin from Engelbreth-Holm-Swarm murine sarcoma basement membrane (Sigma-Aldrich, L2020) and routinely cultured in primary cell medium, composed of Neurobasal (Gibco, 21103-049) and Dulbecco's modified Eagle medium/F12 (Gibco, 31331-028) media (1:1 mix) without serum, supplemented with 1× N2 (Gibco, 17502-048), 1× B27 (Gibco, 12587-010), 10 ng/mL human recombinant FGF2 (Peprotech, 100-18B), 10 ng/mL EGF (Peprotech, AF-100-15), 100 U/mL penicillin and 100 μg/mL streptomycin (PEST; Sigma-Aldrich, P0781). For 3D cultures, GBM cells were seeded at 2 × 10^5^ cells/mL in uncoated dishes, in the same culture medium as described above, and cultured on an orbital shaker at 90 rpm. At 7 to 10 d, spheroids reached a diameter of 0.3 to 0.5 mm, and the presence of a hypoxic core was observed using Image-iT Green Hypoxia Reagent (Thermo Fisher Scientific, I14833). Spheroid and 2D cultures were dissociated using TrypLE express (Gibco, 12605010). All cells were grown in a humidified 5% CO_2_ incubator at 37 °C.

### Patient GBM Specimens.

Clinical specimens were collected from patients referred to the Neurosurgery Department at Lund University Hospital. Inclusion criteria were age 18 y or above, WHO performance status 0 to 2, and ability to give written informed consent. The study was carried out according to the ICH/GCP guidelines, in agreement with the Helsinki declaration, and approved by the local ethics committee, Lund University (Dnr. 2018/37). Patients were diagnosed by routine MRI of the brain and surgical and pathological procedures, received standard oncological treatment, and were followed up according to local and national recommendations. Clinical-pathological characteristics of included patients are given in *SI Appendix*, Table S1. Fresh samples of macroscopically viable tumors were directly processed for TS-MAP or cryopreserved by snap freezing in isopentane for further immunofluorescence (IF) evaluation (see below).

### TS-MAP: Surfaceome and Endocytome Biotinylation.

For surfaceome profiling in 3D and fresh patient samples, we developed a procedure emanating from studies with HeLa cell-line monolayers (49). Subconfluent primary GBM cell 2D monolayers, 0.3- to 0.5-cm diameter spheroids and mouse or patient tumor pieces (0.3 to 0.5 cm) were preincubated on ice for 10 min and maintained on ice to prevent internalization (under 100 rpm orbital agitation for spheroids and tissue samples). Cells and tissues were washed twice with ice-cold phosphate-buffered saline (PBS) containing MgCl_2_ and CaCl_2_ (Mg/Ca–PBS; Thermo Scientific) adjusted to pH 8.0, followed by incubation with 1 mg/mL of a membrane impermeable and cleavable biotin moiety (EZ-Link Sulfo-*N*-hydroxysuccinimide-SS-biotin, Thermo Scientific, 21331) in Mg/Ca–PBS, pH 8.0 for 30 min protected from light, and washed with ice-cold Mg/Ca–PBS. To preclude unspecific cellular entry of the biotinylation reagent or of secreted/extracellular proteins, free biotin was quenched with 0.1 M glycine in Mg/Ca–PBS for 10 min, and cells were subsequently washed twice with ice-cold Mg/Ca–PBS for further processing.

For endocytome profiling, following cell-surface biotinylation, endocytosis was initiated by the addition of prewarmed primary cell medium for the indicated time periods in a humidified 5% CO_2_ incubator at 37 °C. Endocytosis was finalized by incubation on ice for 10 min, and remaining cell-surface biotin was removed with the membrane impermeable, reducing agent, MesNa (200 mM, sodium-2-mercaptoethanesulfonate; Thermo Scientific, M1511), dissolved in 50 mM Tris pH 8.6 containing 100 mM NaCl, 1 mM ethylenediaminetetraacetic acid (EDTA) and 0.2% bovine serum albumin (BSA) twice for 15 min each time at 4 °C in the dark. Samples were then washed with ice-cold Mg/Ca–PBS and incubated with iodoacetamide (5 mg/mL, Sigma-Aldrich, I6125) in Mg/Ca–PBS for 10 min in the dark, and subsequently washed with Mg/Ca–PBS. For LC-MS/MS experiments, an additional cell-surface biotin blocking step was performed prior to cell lysis using unconjugated streptavidin (50 μg/mL; Sigma-Aldrich, S4762) diluted in Mg/Ca–PBS containing 1% BSA for 30 min at 4 °C, followed by extensive washes with 12 mL of PBS 0.1% Triton X-100, followed by 10 mL of radioimmunoprecipitation assay (RIPA)/PBS 0.1% Triton X-100 1M NaCl 1:1 (vol/vol), and finally 10 mL of PBS 0.1% Triton X-100 to remove unbound streptavidin. As a control of cell-surface biotin removal efficiency, we verified the absence of signal after the MesNa and streptavidin blocking steps in surface-biotinylated cells that did not undergo endocytosis.

### Quantification of Surfaceome and Endocytome by FACS.

Biotinylated surfaceome and endocytome (1.5-h endocytosis) from 2D, spheroid, mouse, and patient samples, as indicated, were quantified on a per-cell basis after gentle detachment/dissociation with TrypLE express (Thermo Fisher Scientific). Endocytosis samples were permeabilized for 30 min with 0.5% saponin in PBS. Unspecific binding was blocked with 3% BSA in PBS for 30 min at room temperature (RT). Biotinylated cell labeling was performed with 5 μg/mL streptavidin-Alexa Fluor (AF)-488 (Life Technologies, S32354) in Mg/Ca–PBS containing 3% BSA for 30 min at 4 °C. Data were acquired on an Accuri C6 flow cytometer and analyzed using FlowJo software (version 10). Results are expressed as normalized mean fluorescence intensity (MFI) after subtraction of the values of negative control cells.

### Fluorescence Microscopy.

Biotinylated spheroids and mouse and patient samples were fixed for 15 min in 4% paraformaldehyde (PFA), cryopreserved by overnight incubation in 0.5 M sucrose, and embedded in optimal cutting temperature (OCT) medium (Fisher Scientific, 12678646) for cryosectioning. Alternatively, biotinylated tissue samples were enzymatically dissociated with TrypLE express and DNase I (Thermo Fisher Scientific, 90083), for 10 min at 37 °C to obtain SCSs, which were filtered through 70- and 40-µm nylon cell strainers, and red blood cells (RBCs) were removed using RBC Lysis Buffer (BioLegend, 420301). Biotinylated primary GBM cells growing as 2D monolayer and SCSs from spheroids and tissues were seeded in eight-well chamber slides (Ibidi, 80827), washed with PBS, fixed with 4% PFA for 10 min, and permeabilized for 30 min with 0.5% saponin diluted in PBS. Nonspecific sites were blocked with PBS containing 3% BSA. Cryosections were blocked in PBS with 5% goat serum (Dako, X0907) for 1 h at RT. Biotinylation was visualized by labeling with 5 µg/mL streptavidin-AF-488 (Thermo Fisher Scientific, S32354) in PBS-3% BSA or PBS-5% goat serum for 30 min at 4 °C. IF staining of cells and sections was performed overnight at 4 °C using the following primary antibodies: Rabbit anti-EEA1 (1/200, Abcam, ab2900), rabbit anti-EGFR (1/200, Abcam, ab32198), rabbit anti-CSPG5 (1/200, Thermo Fisher Scientific, PA5-75459), mouse anti-CD63 (1/100, Abcam, ab8219), mouse anti-CD45-PE (1/25, BD Biosciences, 555483), rabbit anti-CD68 (1/800, Cell Signaling Technology, D4B9C), mouse anti-CD31 (1/100, Dako, JC70A), mouse anti-CD44 (1/100, Dako, DF1485), mouse anti-CD14-PE (1/25, BioLegend), rabbit anti-BCAN (1/200, Abcam, ab111719), rabbit anti-CD81 (1/200, Abcam, ab219209), rabbit anti-TF (kindly provided by Wolfram Ruf, Center for Thrombosis and Hemostasis, University of Mainz, Mainz, Germany), or rabbit anti-MCT2 (1/100, Thermo Fisher Scientific, PA5-112712). After washing, cells were incubated with anti-mouse or anti-rabbit AF-488 or AF-546-conjugated secondary antibody (1/500) (Thermo Fisher Scientific, A11001, A11060, A11008, and A11010) for 1 h at 4 °C. Nuclei were counterstained with Hoechst 33342 (1/20,000p) (Thermo Fisher Scientific, 1399) for 10 min. Sections were finally mounted with Fluorescence Mounting Medium (Dako, S3023). In some cases, subconfluent GBM cells were transfected with a plasmid (250 ng) encoding CD63-mCherry (kindly provided by Jennifer Lippincott-Schwartz, Howard Hughes Medical Institute, Ashburn, VA) using Lipofectamine 2000 (Thermo Fisher Scientific) according to manufacturer protocol.

Imaging (fixed and live cells) was performed using a Zeiss LSM 710 confocal microscope equipped with a 34-channel confocal spectral detector or Airyscan array detector unit. Light sources were a diode laser 405–SF30 (405 nm), a Lasos LGK 7812 argon laser (458 nm, 488 nm, and 514 nm), and a HeNe633 laser (633 nm). Plan-Apochromat 20×/0.8, or Plan-Apochromat 40×/1.40 differential interference contrast (DIC) M27 oil immersion or Plan-Apochromat 63×/1.40 DIC M27 oil immersion objectives were used. Images were processed using ZEN 2.1 black edition (Carl Zeiss).

### High-Affinity Chromatography Enrichment of Biotinylated Proteins.

Biotinylated samples were lysed for 20 min at 4 °C in RIPA buffer (150 mM NaCl, 50 mM Tris pH 8.6, 1% Nonidet P-40, 0.1% sodium dodecyl sulfate, 0.05% sodium deoxycholate) containing 2× protease inhibitors (Roche Diagnostics, 04693124001). Lysates were clarified by centrifugation at 18,000 × *g* for 10 min at 4 °C. The soluble fraction was collected, and total protein was quantified using BCA Protein assay kit (Pierce, 23225). Lysates were diluted 1:4 with Mg/Ca–PBS supplemented with protease inhibitors, filtered with a 0.45-µm surfactant-free cellulose acetate syringe filter, and then applied to a HiTrap streptavidin HP-1 mL column (GE Healthcare, 17-5112-01) pre-equilibrated in PBS 0.1% Triton X-100 using a peristaltic pump set at a flow rate of 250 μL/min. The column was then transferred to an high-pressure liquid chromatography (HPLC) UPC 900 system (Amersham Biosciences) equipped with an online ultraviolet (UV) detector set at 280 nm, and washed with 10 mL of PBS 0.1% Triton X-100, followed by 10 mL of RIPA/PBS 0.1% Triton X-100 1 M NaCl 1:1 (vol/vol), and finally 10 mL of PBS 0.1% Triton X-100 to remove nonbiotinylated proteins at a flow rate of 1 mL/min. Biotinylated proteins were then eluted from the column by reduction of the protein-SS-biotin linker with 10 mL freshly prepared 150 mM MesNa in PBS 0.1% Triton X-100 applied at a reduced flow rate (125 μL/min). One volume of 20% trichloroacetic acid was added to the collected eluate to precipitate proteins by incubation for 30 min on ice and centrifugation for 10 min at 18,000 × *g*. Protein pellets were finally washed twice with 2% sodium acetate and resuspended in 6 M urea buffer for LC-MS/MS sample preparation.

### Sample Preparation for LC-MS/MS.

Protein pellets, isolated as described above, were dissolved in 50 mM ammonium bicarbonate (AMBIC) buffer containing 6 M urea (5× the sample volume). Disulfide bonds within protein samples were then reduced by incubating samples with 10 mM dithiothreitol for 1 h at 56 °C at 300 rpm and alkylation of free sulfhydryl groups was done with 30-min incubation with 20 mM iodoacetamide in the dark at RT, followed by buffer exchange with 50 mM AMBIC, pH 8.0. Protein samples were digested with 0.4 μg/μL of sequencing grade porcine trypsin (Promega, V511A) in AMBIC at an enzyme-to-protein relation of ∼1:100 overnight at 37 °C under 350 rpm agitation. The digestion was stopped by adding 1.73% trifluoroacetic acid (TFA) and samples were dried using a Speed Vac, resuspended in 100 µL of 0.1% TFA in dH_2_0 and desalted with Ultra MicroSpin silica C18 columns (The Nest Group, SUM SS18V). Samples are eluted with a 50% acetonitrile (ACN), 0.1% TFA solution in dH2O by spinning at 200 × *g* for 1 min and then dried with the Speed Vac and stored at −80 °C until further analysis.

### Mass Spectrometry Analysis.

LC-MS/MS analysis was performed on a Tribrid Mass Spectrometer Fusion (Thermo Fisher Scientific) equipped with a Nanospray Flex ion source and coupled with an EASY-nLC 1000 Ultra high pressure liquid chromatography (UHPLC) pump (Thermo Fisher Scientific). For the analysis, samples were resuspended in 2% ACN-0.1% TFA, and peptide concentration was adjusted to 0.5 µg/µL by measuring 215 nm absorbance using a DeNovix DS-11 spectrophotometer, and 1 µg of peptides was injected in one to three independent runs depending on total sample peptide amount. Peptides were concentrated on an Acclaim PepMap 100 C18 precolumn (75 μm × 2 cm, Thermo Fisher Scientific) and then separated on an Acclaim PepMap RSLC column (75 μm × 25 cm, nanoViper, C18, 2 μm, 100 Å) set at 45 °C and a flow rate of 300 nL/min. Solvent A (0.1% formic acid in water) and solvent B (0.1% formic acid in acetonitrile) were used to create a nonlinear elution gradient. The Orbitrap Fusion was operated in the positive data-dependent acquisition (DDA) mode. The peptides were introduced into the LC-MS via a stainless steel nano-bore emitter (outer diameter [OD] 150 µm, inner diameter [ID] 30 µm) with the spray voltage of 2 kV and the capillary temperature set at 275 °C. Full MS survey scans from *m/z* 350 to 1,350 with a resolution of 120,000 were performed in the Orbitrap detector. The automatic gain control (AGC) target was set to 4 × 10^5^ with an injection time of 50 ms. The most intense ions (up to 20) with charge states 2 to 5 from the full scan MS were selected for fragmentation in the Orbitrap. The MS2 precursors were isolated with a quadrupole mass filter set to a width of 1.2 *m/z*. Precursors were fragmented by high-energy collision dissociation (HCD) at a normalized collision energy (NCE) of 30%. The resolution was fixed at 30,000 and for the MS/MS scans, the values for the AGC target and injection time were 5 × 10^4^ and 54 ms, respectively. The duration of dynamic exclusion was set to 45 s and the mass tolerance window was 10 ppm. For comparison, some samples were analyzed on a Thermo Easy nLC 1000 system (Thermo Fisher Scientific) coupled online to a Q-Exactive HF-X mass spectrometer (Thermo Scientific). Peptides (1 µg) were loaded on a precolumn (Thermo Scientific; ID 75 μm × 2 cm, temperature 35 °C) and then separated on an EASY-Spray column (Thermo Scientific; ID 75 μm × 25 cm, temperature 45 °C). A nonlinear gradient of buffer B (80% acetonitrile, 0.1% formic acid) in buffer A (aqueous 0.1% formic acid) was applied at a flow rate of 300 nL/min. One full MS scan (resolution 120,000 @ 200 *m/z*; mass range 375 to 1,500 *m/z*) (resolution 15,000 @ 200 *m/z*). The precursor ions were isolated with 1.2 *m/z* isolation width and fragmented using higher-energy collisional-induced dissociation at a normalized collision energy of 28. Charge state screening was enabled, and singly charged ions as well as precursors with a charge state above 6 were rejected. The dynamic exclusion window was set to 40 s. The automatic gain control was set to 3 × 10^6^ for MS and 1 × 10^5^ for MS/MS with ion accumulation times of 50 ms and 19 ms, respectively. The intensity threshold for precursor ion selection was set to 5.3 × 10^5^.

The raw DDA data were analyzed with Proteome Discoverer 2.3 (PD 2.3) Software (Thermo Fisher Scientific). Peptides were identified using SEQUEST HT against UniProtKB human database (release 2020_05), using the following parameters: Static modification, cysteine carbamidomethylation and dynamic modifications, N-terminal acetylation and methionine oxidation. Precursor tolerance was set to 10 ppm and fragment tolerance to 0.05 ppm. Up to two missed cleavages were allowed and Percolator was used for peptide validation at a *q* value of maximum 0.05. Protein abundance was calculated based on intensity of unique peptides after low abundance resampling missing value imputation and total peptide amount normalization.

### SURFME Classifier for Filtering of Bona Fide Cell-Surface Proteins.

An in-house list of curated protein IDs belonging to the human surfaceome was generated. An initial set of plasma membrane–associated proteins was constructed by merging nine terms from public databases: 2,886 proteins predicted by SURFY (retrieved from http://wlab.ethz.ch/surfaceome/) (1); 5,196 listed in plasma membrane GO term (GO:0005886); 935 in cell surface GO term (GO:0009986); 437 in GO’s external side of plasma membrane term (GO:0009897); 939 reviewed UniProt GPI-anchored proteins; 2,356 reviewed UniProt single-pass transmembrane proteins; 2,819 reviewed UniProt multipass transmembrane proteins; 3,724 reviewed UniProt cell membrane proteins; and 2,726 reviewed UniProt proteins within extracellular domain topological term. Duplicates and false positives were filtered out by curation based on further database annotation (from UniProt, GO, and GeneCards) and literature review (PubMed.gov). We finally identified a total of 3,317 proteins, hereafter defined as SURFME (see also *Results* section and Dataset S1).

### Softwares, Bioinformatics Tools, and Statistical Analyses.

Human homologs to mouse symbols were obtained with the BiomaRT package. The 135 mouse symbols not mapped to human equivalents by BiomaRT were manually searched using mouse genome informatics homology information (http://www.informatics.jax.org/). Bioinformatics analyses were conducted in R version 4.0.4 and figures were generated using the packages ggplot2, RColorBrewer, viridis, VennDiagram, venneuler (in combination with http://bioinformatics.psb.ugent.be/cgi-bin/liste/Venn/calculate_venn.htpl to perform six-group overlap comparison), and pheatmap (clustering method used was “ward.D2”). GraphPad Prism 8.3.1 was used to generate some of the bar plots, and graphical illustrations were created with BioRender.com. Figure design and composition were performed with Adobe Illustrator 25.2. Statistical analyses were performed in GraphPad Prism using two-way ANOVA with Tukey’s post hoc test for multiple comparison. All values with *p* < 0.05 were considered statistically significant.

## Supplementary Material

Supplementary File

Supplementary File

Supplementary File

Supplementary File

Supplementary File

Supplementary File

## Data Availability

All study data are included in the article and/or supporting information.
