# Supplementary material for: Landscape of surfaceome and endocytome in human glioma is divergent and depends on cellular spatial organization
Source: Proc Natl Acad Sci U S A. 2022 Feb 25;119(9):e2114456119. doi: 10.1073/pnas.2114456119 (PMC8892282; doi:10.1073/pnas.2114456119)
Supplement: Supplementary File [file pnas.2114456119.sapp.pdf]

Landscape of surfaceome and endocytome in human glioma is divergent and depends on cellular spatial organization

Valeria Governa[a,1], Hugo Talbot[a,1], Kelin Gonçalves de Oliveira[a,1], Myriam Cerezo-Magaña[a], Anna Bång-Rudenstam[a], Maria C Johansson[a], Ann-Sofie Månsson[a], Karin Forsberg-Nilsson[b], György Marko-Varga[c,d,e], Julio Enríquez Pérez[f], Anna Darabi[f], Johan Malmström[g], Johan Bengzon[f,h], Charlotte Welinder[a], Mattias Belting[a,b,i,2]

[1]These authors contributed equally

[2]To whom correspondence should be addressed: Mattias Belting, Department of Clinical Sciences, Lund, Section of Oncology, Lund University, Barngatan 4, SE-221 85, Lund, Sweden; Tel: +46-46-178549; E-mail: mattias.belting@med.lu.se

SUPPLEMENTARY METHODS

Mouse GBM model. Experimental procedures were approved by the ethical committee for animal research in Malmö/Lund (ethical permit M144-14 and M145-14), and performed in accordance with the European Union animal rights and ethics directives. GL261 mouse glioma cells of C57BL/6 origin (22) were expanded in DMEM supplemented with 2 mM L-glutamine, 1 mM sodium pyruvate, 10 mM HEPES, 50 µg/mL gentamicin (GIBCO-Life technologies) and 10% fetal bovine serum (FBS; Biochrom AG). Cells were kept subconfluent, detached in trypsin-EDTA, and allowed to recover in DMEM with 10% FBS for 20 minutes during counting/viability measurements. GL261 cells were then washed twice and finally resuspended in serum-free (SF) DMEM without antibiotics. For orthotopic injections, 8 weeks old syngeneic, female C57BL/6 mice (Scanbur) were anaesthetized with isoflurane and placed on a stereotactic frame. Mice received Visco-tears and local anesthetic *s.c.* (Marcaine), a hole was drilled in the skull and 5 µL of the cell suspension (50.000 cells) was slowly injected 1.5 mm to the right and 1.0 mm anterior of bregma, 2.5 mm deep from the dural surface using a Hamilton syringe. The needle was left in place for 2 minutes, and slowly retracted before the skull hole was filled with bone wax and the wound closed with clips. Mice finally received Temgesic analgesic (0.3 mg/mL) *s.c.* in the posterior leg. Mice were monitored daily and sacrificed when the first mouse in the experiment presented neurological symptoms (33 days). Mouse brains were collected, and separated into "Normal brain" and "Brain tumor" (non-tumor tissue was dissected out) and directly processed for TS-MAP or snap-frozen in isopentane before storage at -80ºC and sectioning for histological analyses.

Western Blotting. Cell lysis was performed in RIPA buffer (150 mM NaCl, 50 mM Tris pH 7.4, 1% NP-40, 0.1% SDS, 0.05% sodium deoxycholate) and protein concentration was assessed by using BCA Protein assay kit (Pierce, 23225). Protein lysates were mixed with

NuPAGE 4 × LDS Sample Buffer (Invitrogen, NP0007) and heated for 10 minutes at 80 °C. Biotinylated surface proteins, biotinylated endocytosed proteins and control samples (10 μg) were separated by electrophoresis using a NuPAGE 4–12% Bis Tris gel (Invitrogen, NP0321PK2) in non-reducing conditions using SeeBlue Plus2 (Invitrogen, LC5925) as molecular mass standard. Proteins were then electroblotted to a polyvinylidene fluoride (Immobilon-FL PVDF) transfer membrane (Merck Millipore, IPFL00010), followed by blocking in TBS containing 0.05% Tween 20, 3% BSA for 1h at RT. To probe for biotin, membrane was incubated with Streptavidin HRP-linked (Thermo Scientific, N100) (1:2000) in TBST containing 3% BSA for 1 h at RT. Protein bands were visualized by Pierce enhanced chemiluminescence (ECL) western blotting substrate (Thermo Scientific, 32209).

Ligand uptake. Surface-biotinylated cells were incubated with 5 μg/ml Cholera Toxin subunit B-Alexa-647 (ThermoFisher Scientific, C34778), or 500 μg/ml Rhodamine B isothiocyanate Dextran 70 kDa (Sigma-Aldrich, R9379) for 0.5h or 2h at 37 °C in primary cell medium. Ligand uptake and endocytosis was stopped by putting cells on ice for 10 minutes and remaining surface biotin was removed by MesNa. Cells were then fixed in 4% PFA for further fluorescence imaging.

Biotinylation of PBMCs and CD14+ MACS sorting. Blood buffy coats were obtained from patients or healthy individuals (the Transfusion Medicine service, Skåne University Hospital, Lund), and PBMCs were isolated by density-gradient centrifugation on Ficoll® Paque Plus (GE Healthcare, 17-1440-02) at 700 rpm for 20 minutes. Surface biotinylation of PBMCs was carried out as with GBM cells. Isolation of the $CD14^+$ population was performed with CD14 human MicroBeads (Miltenyi Biotec, 130-050-201) and magnetic MS columns (Miltenyi Biotec, 130-042-201) according to manufacturer's instructions. Cells were fixed in 4% PFA for

10 minutes and blocked with 3%BSA in PBS for 30 minutes. Staining was done using 5 µg/mL Streptavidin-AF-488, CD14-APC (BioLegend, 325607) and CD45-PE (BD, 555483). After staining, analysis was performed by confocal microscopy or FACS using FACSCalibur™ (BD Biosciences).

Incucyte Cytotox assay. U3065 cells were seeded at a density of 10^4 cells per well in a 96-well round bottom plate (Sigma-Aldrich, BR781900) 72h before the start of the assay to allow single spheroid formation (around 400 µm diameter). For 2D monolayer, U3065 cells were seeded in a laminin-coated 96-well plate at a density of 10^4 cells the day before the start of the assay. Treatment was initiated by addition of 0.5 to 5 nM of anti-EGFR antibody (Abcam, ab52894) pre-complexed with equal amount of anti-rabbit IgG Fc-monomethyl auristatin F (αOFc-NC-MMAF) secondary ADC (Moradec LLC, AO-101AF-50). At the same time 250 nM of Incucyte® Cytotox Red Dye (Essen Bioscience, 4632) was added to follow cell death. In the control condition, the same amount of Incucyte® Cytotox Red Dye and αOFc-NC-MMAF equivalent to the highest anti-EGFR concentration but without primary antibody was used. Live image acquisition was done every 6h for 48h with 4x (U3065 3D) and 10x (U3065 2D) objectives of an Incucyte® S3 system placed in a humidified 5% CO_2 incubator at 37 °C. Data are expressed as the mean ± S.D. Statistical analyses were performed in GraphPad Prism using Two-Way ANOVA with Tukey post hoc test for multiple comparison. All values with $P < 0.05$ were considered statistically significant.

GSEA and pathway analysis of LC-MS/MS data. For enrichment analysis, normalized protein abundances from LC-MS/MS experiments and SURFME set were entered into GSEA software (Broad Institute / UC San Diego, version 4.1.0) as Expression dataset and Gene sets database, respectively. Ratio of classes between Surface and Control samples or Endocytosed

and Control samples was performed as gene ranking metrics. Permutation type was set to gene_set. Chip platform was Human_UniProt_IDs_MSigDB.v7.4.chip. Maximum set size was set to 4000. Mode chosen was Sum_of_probes and the collapsing parameter was set to No_Collapse. Other parameters were left as advised by the program's default configurations. For pathway analysis, Gene symbols (HGNC IDs) of proteins overexpressed in U3065 3D compared to 2D, and mouse GL261 GBM compared to normal brain (gene symbols transformed to human homologs, see below) were obtained and used as input for over-representation analysis tool under gene set analysis tab available in CPDB's website (http://cpdb.molgen.mpg.de/). Pathway-based set (Pathways as defined by pathway databases) was used, including KEGG, Reactome and PID. Minimum overlap with input list was set to 4 and p-value cutoff was set to 0.001.

SUPPLEMENTARY FIGURES AND LEGENDS

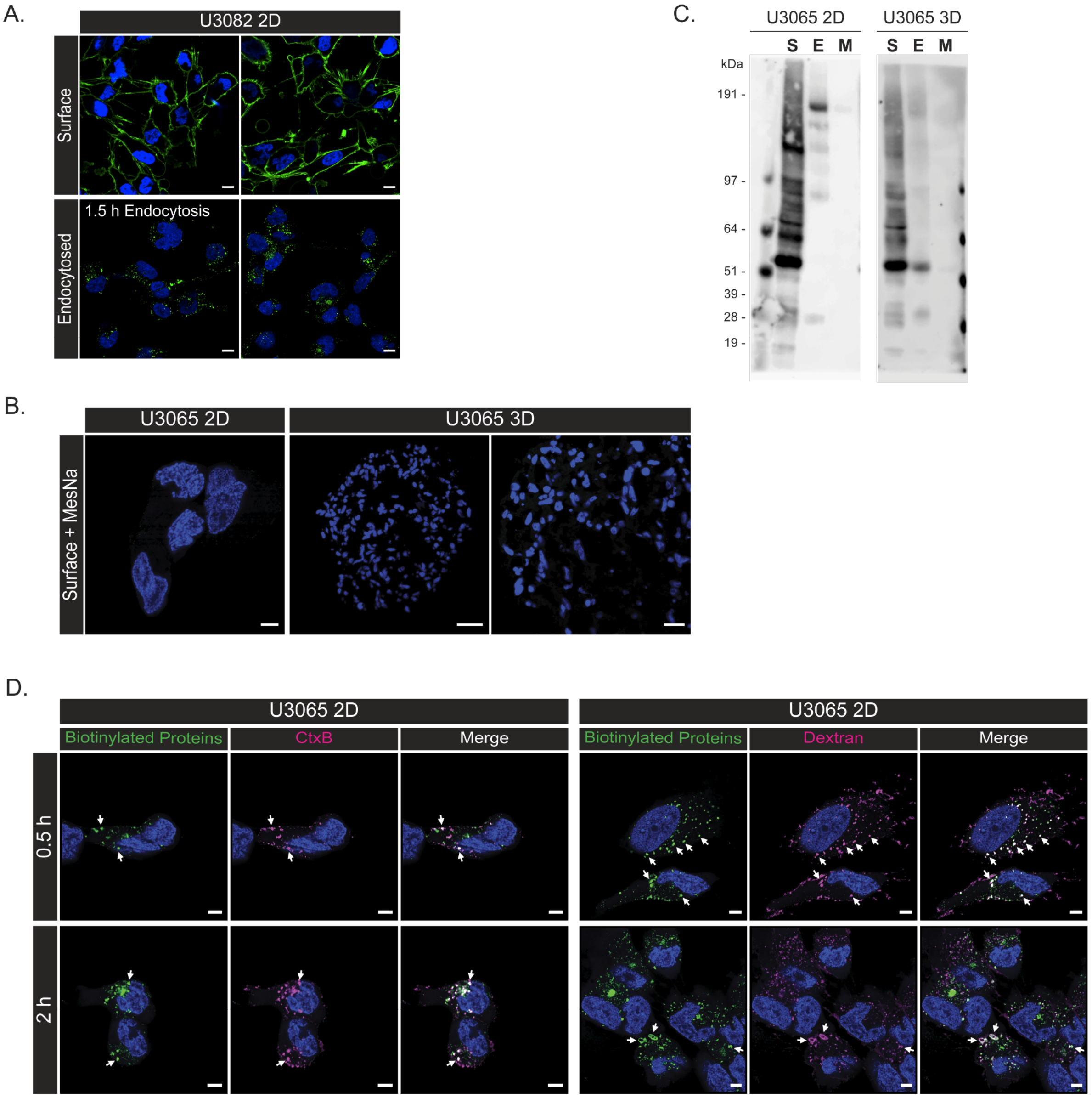

Figure S1. A) Confocal microscopy imaging of surface (upper panels) and endocytosed (lower panels) biotinylated proteins (green) in U3082 GBM cells grown in 2D. Scale bars, 10 µm. **B)** Confocal microscopy imaging of surface biotinylated proteins (green) after biotinylation removal by MesNa in U3065 GBM cells grown in 2D or 3D, as indicated. Scale bars, 5 µm (2D), 50 µm (3D, left panel) and 20 µm (3D, right panel). **C)** Western blotting of biotinylated proteins shows efficient surfaceome (S) and endocytome (E) labeling, and removal by MesNa (M) in U3065 from 2D and 3D cultures. **D)** Confocal microscopy imaging of endocytosed

biotinylated proteins (green) and fluorescently labelled ligands CtxB and Dextran (red). Endocytosis and ligand uptake occurred concomitantly for 0.5 or 2 h in U3065 GBM cells grown in 2D. White arrows indicate co-localization. Scale bars, 5 μm.

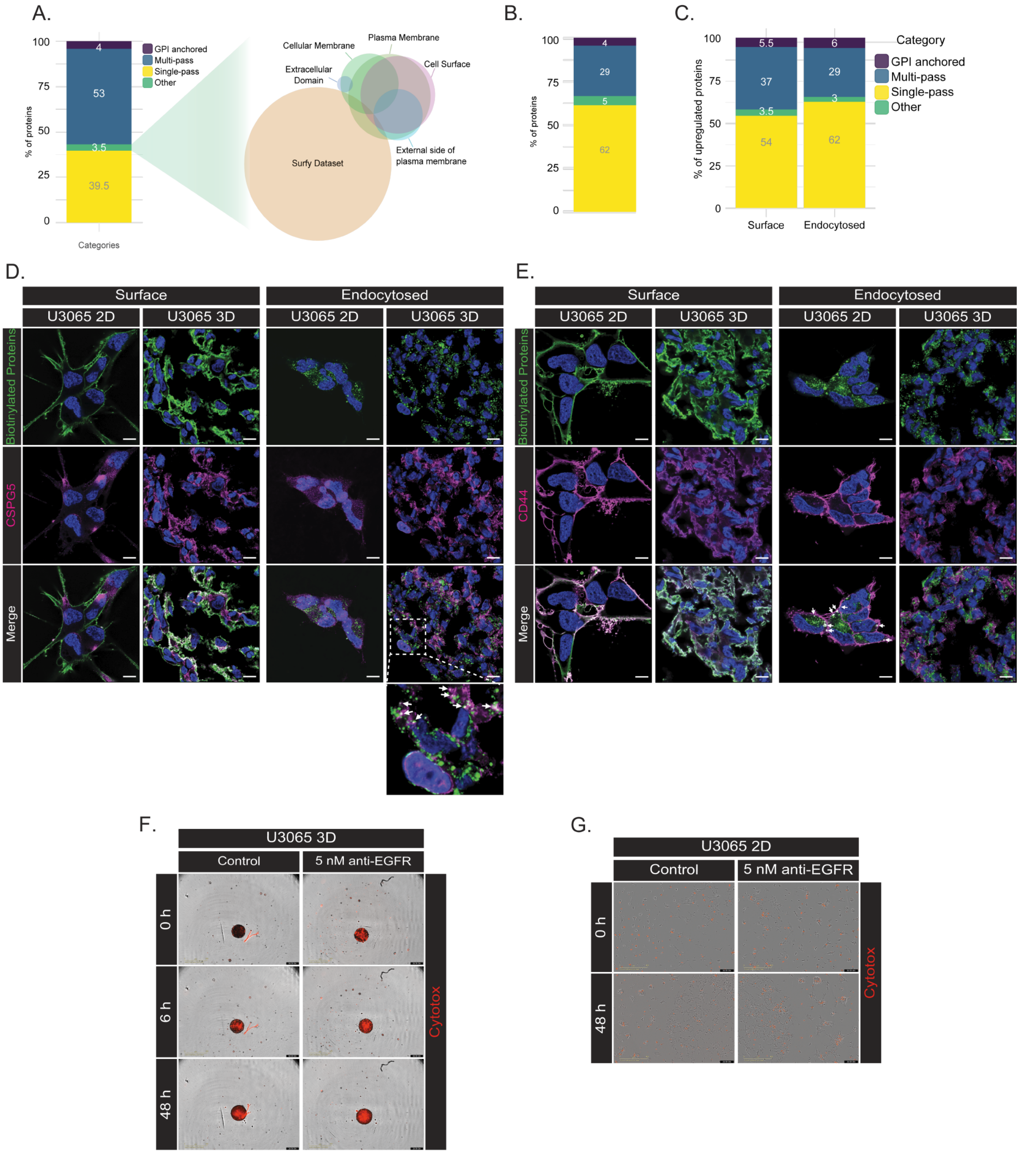

Figure S2. A) Stacked bar chart representing categories of protein identities in the SURFME classifier. The main categories are Multi-pass, Single-pass transmembrane and GPI-anchored proteins. Venn diagram describes annotation of SURFME protein identities not belonging to these three categories. **B)** Stacked bar chart representing protein categories of SURFME proteins identified in U3065 samples. **C)** Stacked bar chart suggests relative enrichment of multi-pass transmembrane proteins among surface proteins upregulated in 3D *vs.* 2D (log_2FC

≥ 0.5). **D)** and **E)** Immunofluorescence validation: 2D and 3D cultures, as indicated, were surface biotinylated and allowed to endocytose (Endocytosed proteins) or not (Surface proteins). 2D cells and 3D cryosections were then stained with streptavidin-AF488 (green) and anti-CSPG5 (**D**) or anti-CD44 (**E**) antibody (magenta). White arrows indicate co-localization. Scale bars, 10 μm. **F, G)** Live imaging of U3065 spheroids **(F)** or cultured in 2D **(G)**, treated or not with anti-EGFR ADC at the indicated time-points. Cytotoxicity is visualized by Cytotox-Red fluorescent probe. Scale bars appear at the bottom right of each image, 800 μm **(F)** and 400 μm **(G)**.

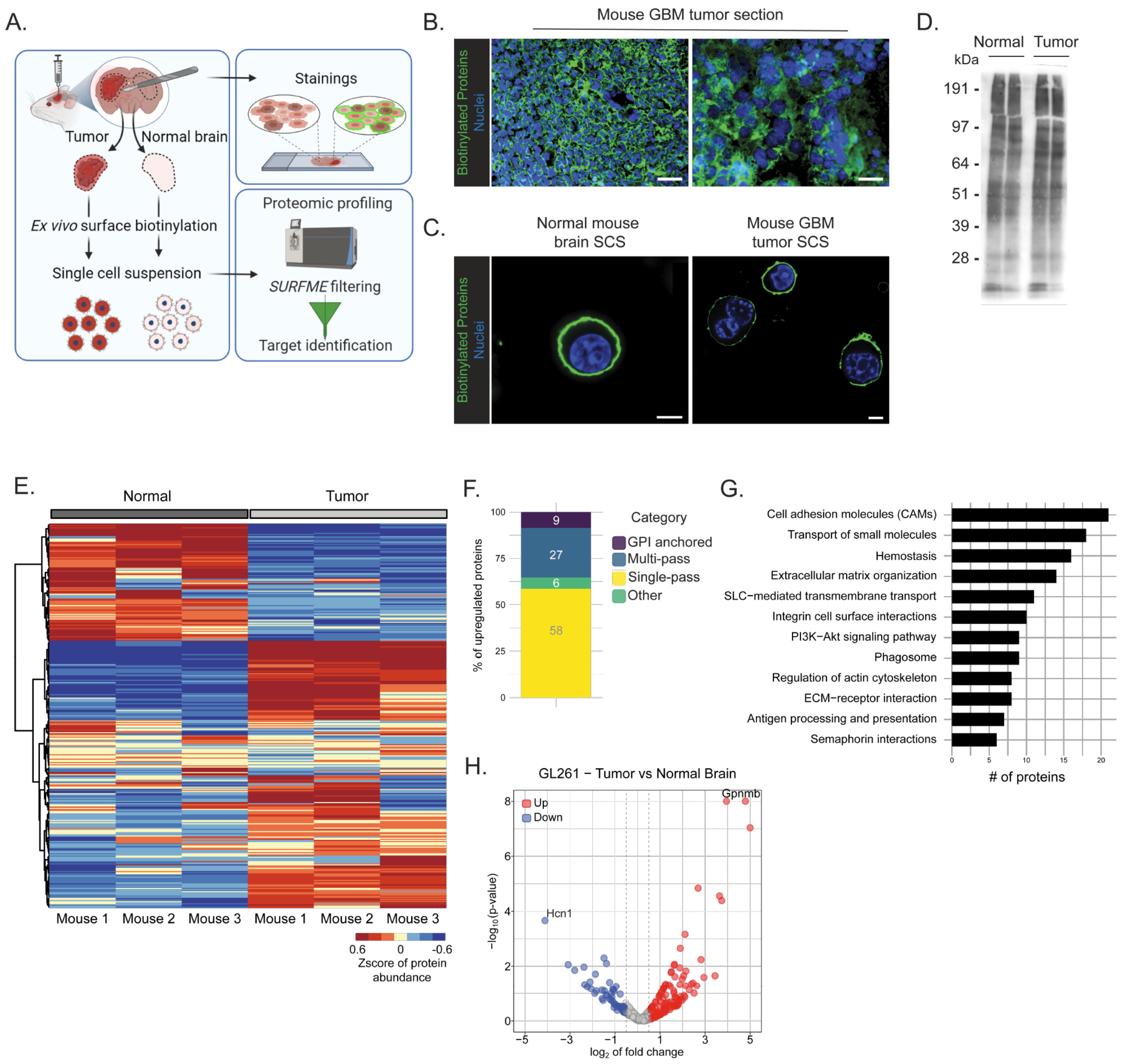

Figure S3. Tumor surfaceome mapping (TS-MAP) of mouse GBM and non-tumor brain tissue. **A)** Orthotopic mouse GBM tumors and non-tumor tissue of the contralateral hemisphere were dissected and biotinylated *ex vivo* for downstream analyses, as indicated. **B)** Immunofluorescence microscopy shows biotinylation (green) of intact mouse tumor. Higher magnification (right panel) indicates surface labelling, which is further supported by Airyscan imaging of single cell suspension (SCS) of disintegrated tissue (**C**), showing specific plasma membrane labelling in both tumor and non-tumor tissue. Shown are representative images from at least 3 repeat experiments. Scale bars, 50 µm (B, left), 20 µm (B, right), and 5 µm (C). **D)** Western blotting shows comparable surfaceome biotinylation in intact tumor and non-tumor

tissue from two technical repeat experiments. **E)** Surfaceome protein abundance heatmap demonstrates divergent SURFME protein expression in tumor *vs.* non-tumor tissue. **F)** Stacked bar chart of SURFME protein categories upregulated (≥0.5 log_2 FC) in tumor *vs.* non-tumor tissue. **G)** Selection of overrepresented pathways for SURFME proteins upregulated in tumor *vs.* non-tumor tissue. **H)** Volcano plot displays magnitude of SURFME protein changes (log_2 fold) *vs.* statistical significance (*p*-value) in tumor compared with non-tumor tissue.

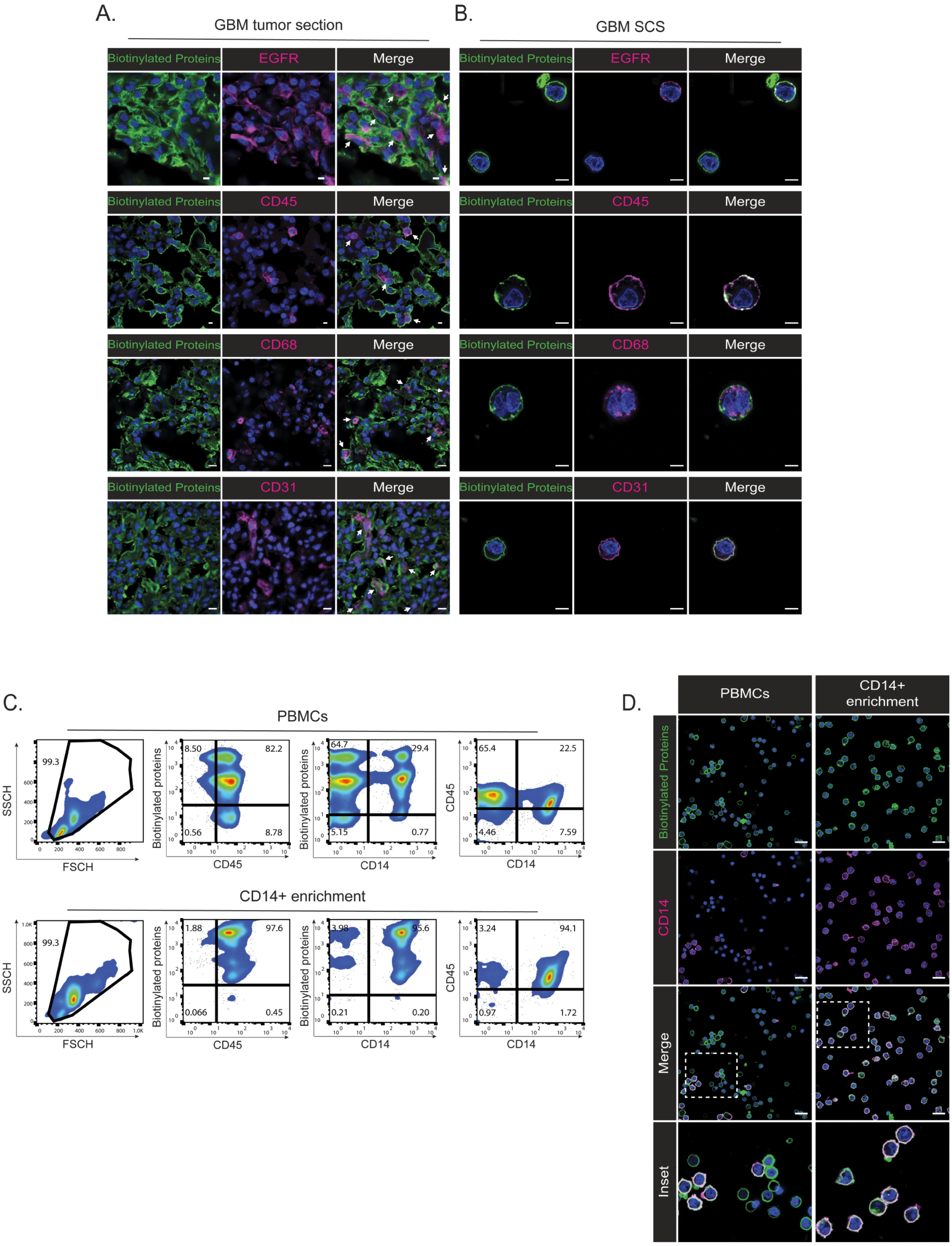

Figure S4. A) Immunofluorescence microscopy of tumor cryosection shows comprehensive cell-surface biotinylation (green) of various tumor cell types (magenta), *i.e.* tumor cells (EGFR), leukocytes (CD45), macrophages (CD68), and endothelial cells (CD31). Scale bars, 5 µm (EGFR and CD45) and 10 µm (CD68 and CD31). **B)** Similar experiment as in (**A**) following

tissue disintegration into SCS, and analyzed by confocal microscopy. Scale bars, 5 μm. **C)** Healthy donor peripheral blood mononuclear cells (PBMCs) were isolated for cell-surface biotinylation, co-stained with streptavidin-AF488 and anti-CD45 or anti-CD14 antibody, and analyzed by FACS. Lower panel: Similar experiment following enrichment for CD14+ monocytes. **D)** Similar experiment as in (**C**) analyzed by confocal microscopy for visualization of specific cell-surface labelling of PBMCs and CD14+ monocytes. White square indicates zoomed area shown in inset. Scale bars, 20 μm. Shown are representative images and FACS plots from at least 3 independent experiments.

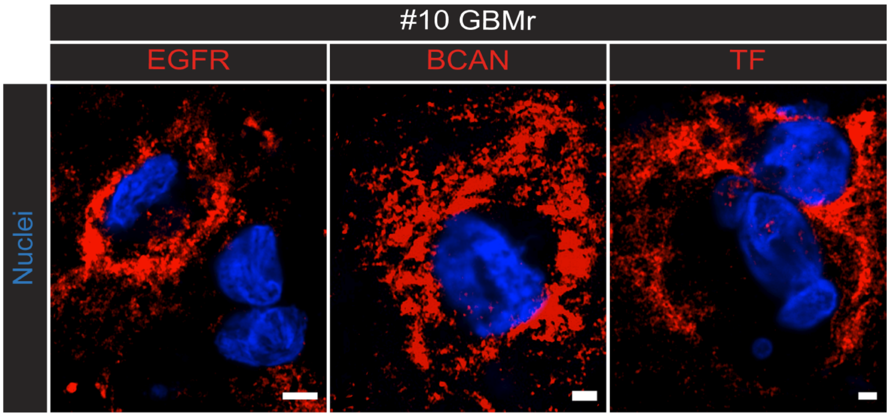

Figure S5. Immunofluorescence validation of LC-MS/MS data in #10GBMr tumor sections shows cell-surface expression and cytoplasmic (vesicular) expression of selected SURFME proteins (red; EGFR, BCAN and TF). Scale bars, 2 µm.

Supplementary Video 1. U3082 cells transfected with CD63-mCherry were surface-biotinylated. Endocytosis was induced 45 minutes prior to start of live imaging acquisition. Live acquisition was performed on a confocal coupled with Airyscan detector capturing one image every 11 seconds for 27 minutes. Video shows imaging of CD63 and biotinylated cell-surface proteins.

Supplementary Video 2. Same experiment as in Supplementary Video 1, showing only biotinylated protein endocytosis, zoomed in on the top right part of the same cell as shown in Supplementary Video 1.

Table S1. Clinicopathological characteristics of TS-MAP glioma cohort.

	Characteristics						
**Patient #**	Age at diagnosis	Gender	Histology	WHO Tumor grade	MGMT status*	IDH1 status	Primary or recurrent
1	61	Female	GBM	IV	Positive	WT	Primary
2	64	Female	GBM	IV	Positive	WT	Primary
3	36	Female	AA	III	Positive	WT	Primary
4	61	Male	Gliosarcoma	IV	Positive	WT	Primary
5	73	Male	GBM	IV	Negative	WT	Primary
6	45	Male	GBM	IV	Negative	WT	Primary
7	67	Female	ODG	II	Positive	Mutant	Primary
8	52	Female	ODG	II	ND	Mutant	Recurrent
9	29	Male	GBM	IV	Positive	Mutant	Recurrent
10	54	Male	GBM	IV	Negative	WT	Recurrent

* O^6-methylguanine-DNA methyltransferase (MGMT) promotor methylation.

AA, anaplastic astrocytoma; GBM, glioblastoma; IDH1, isocitrate dehydrogenase 1; ND, not determined; ODG, oligodendroglioma; WT, wildtype.

Table S2. Surfaceome and endocytome relative abundance ranking of SURFME proteins identified in #10GBMr

Symbol	Surfaceome abundance rank	Endocytome abundance rank
NCAM1	1	2
ATP1A3	2	1
CNTN1	3	8
MOG	4	14
SIRPA	5	21
ICAM5	6	22
IGSF8	7	5
NFASC	8	19
MAG	9	17
ATP1A1	10	4
NCAM2	11	23
THY1	12	39
L1CAM	13	16
CADM3	14	6
ATP1A2	15	3
LSAMP	16	9
CNTNAP1	17	12
SLC1A2	18	7
PLP1	19	28
HEPACAM	20	62
EGFR	21	15
OPCML	22	30
ATP1B1	23	24
PLXNA1	24	20
HSPA8	25	18
SLC3A2	26	36
CADM4	27	61
NRCAM	28	50
NEGR1	29	48
CADM2	30	72
CNTNAP2	31	69
ATP1B2	32	42
NTM	33	49
NPTN	34	58
DPP6	35	37
ATP2B4	36	11
ALCAM	37	65
SLC1A3	38	41

SCN2A	39	89
PLXNA4	40	55
LRP1	41	44
CNTN2	42	60
SLC44A1	43	71
SV2A	44	13
HSPA5	45	35
HSPA2	46	46
LINGO1	47	43
ADAM23	48	76
GPR158	49	96
ATP2B1	50	25
ATP2B2	51	34
CHL1	52	63
ITGAV	53	51
NECTIN1	54	103
PLXNB2	55	92
SLC8A2	56	33
IGSF21	57	83
F3	58	99
PTPRS	59	53
EPHA4	60	80
SLC4A4	61	40
P2RX7	62	32
GRIA2	63	29
LY6H	64	95
ITGB1	65	118
JAM3	66	126
C3	67	64
ADAM10	68	27
ADAM22	69	90
PTGFRN	70	66
DPP10	71	68
PLXNB1	72	127
BTN2A1	73	115
HSP90AA1	74	56
FOLH1	75	116
GRM2	76	77
GRIA3	77	45
BCAN	78	86
ENPP6	79	144
CADM1	80	205

CD59	81	120
GRM5	82	85
GRM3	83	93
PTPRF	84	119
PLXNC1	85	108
OMG	86	237
FAM171A2	87	26
CA4	88	137
TTYH1	89	98
LYNX1	90	79
CFL1	91	125
TMEM30A	92	38
SLC7A5	93	47
RPN1	94	111
GPM6A	95	148
NTRK2	96	73
PTPRD	97	110
GJA1	98	94
HSP90AB1	99	57
HLA-B	100	107
ICAM1	101	131
FAM171B	102	84
CD44	103	155
BTN3A3	104	106
PTPRZ1	105	78
CD38	106	52
PCDH1	107	214
P4HB	108	113
GABBR1	109	228
ASTN1	110	122
ITGB8	111	117
CD200	112	245
ITGA6	113	154
BSG	114	159
SLC12A5	115	59
MCAM	116	211
AQP4	117	75
EPHB2	118	153
LRRC8A	119	133
NPTXR	120	87
NEO1	121	129
OPALIN	122	191

SLC30A1	123	141
HLA-A	124	186
NRXN1	125	130
JAM2	126	207
GPM6B	127	151
TFRC	128	145
ADGRL1	129	101
ATP2B3	130	67
GRIA1	131	82
CD47	132	160
ITGB3	133	31
PLXND1	134	188
ITGA2	135	177
F2	136	109
PLXNA2	137	203
TRPV2	138	81
SLC17A7	139	70
CNTFR	140	166
SLC12A2	141	139
ANXA1	142	128
CSPG5	143	226
KIAA1549L	144	105
ITGB4	145	162
GABBR2	146	124
SLC8A1	147	142
CSPG4	148	216
SLC32A1	149	54
HCN2	150	135
GRM7	151	134
CXADR	152	199
HLA-C	153	190
NTRK3	154	100
SV2B	155	91
SLC44A2	156	164
SLC2A13	157	102
KCNMA1	158	193
GLRB	159	253
BCAM	160	261
ANXA5	161	132
FAM171A1	162	185
CACHD1	163	238
NCSTN	164	197

CNTNAP5	165	201
ITFG1	166	192
ITGB2	167	161
HLA-E	168	287
LRIG1	169	121
EPHB6	170	171
GRIN1	171	175
SLC2A3	172	97
SIRPB1	173	281
MPZ	174	263
FAM234A	175	187
SLC39A10	176	277
SLC39A14	177	251
SLC24A2	178	195
SLC1A4	179	143
SLC9A1	180	112
GRIN2B	181	200
NT5E	182	156
NRXN3	183	158
LRRC4B	184	183
PRNP	185	182
SLC6A1	186	184
SEMA4B	187	194
CDH13	188	114
BMPR2	189	224
GRM1	190	165
TTYH3	191	189
GGT7	192	136
ITGA3	193	235
SEZ6L2	194	225
STX1A	195	206
ITGA7	196	163
DDR1	197	209
ANXA2	198	213
SCN2B	199	270
CA14	200	252
TENM4	201	219
PRRT3	202	104
GABRA1	203	236
PTPRT	204	255
IGSF1	205	231
DRD1	206	295

IGSF9B	207	220
IL6ST	208	288
ADAM9	209	218
PTPRN	210	179
APP	211	181
PTPRN2	212	123
IGF1R	213	227
ENTPD2	214	229
ATP8A2	215	196
CNR1	216	140
SLC17A6	217	167
EVI2A	218	262
CD99L2	219	243
GPRC5B	220	176
RTN4R	221	150
ATP1B3	222	138
GABRG2	223	217
CD14	224	168
SLC20A2	225	222
CACNG3	226	173
CPM	227	157
CLDN11	228	198
PTPRJ	229	240
KCNA2	230	215
COMT	231	149
ERMP1	232	283
NECTIN2	233	267
SORT1	234	250
SLC15A2	235	244
NGFR	236	290
INSR	237	247
ITGAM	238	178
NLGN2	239	172
SHISA4	240	296
MYADM	241	271
CDH2	242	266
KIT	243	282
SYNPR	244	221
PTPRA	245	241
TMX4	246	249
ASTN2	247	273
ADAM17	248	294

GABRA5	249	234
HLA-DRB1	250	269
SLC6A9	251	259
IL13RA1	252	174
ANO6	253	180
ACE	254	276
ATRN	255	256
ROBO2	256	289
SLC4A10	257	204
EPHB3	258	223
SORCS3	259	284
SV2C	260	230
CYBB	261	285
MFSD4A	262	210
CACNG8	263	248
BGN	264	293
SCARF2	265	74
GABRB3	266	208
SLC16A7	267	258
CDH20	268	170
SLC6A11	269	88
MELTF	270	291
SEMA4D	271	292
ADGRL3	272	242
PI16	273	278
SLC39A6	274	279
SLC4A1	275	275
AQP1	276	268
UNC5C	277	246
GABRA3	278	274
ASIC1	279	212
ADCYAP1R1	280	280
AZGP1	281	169
NRXN2	282	265
TMEM245	283	147
TTYH2	284	257
SLC38A3	285	239
TYRO3	286	202
LPAR1	287	297
KCNA1	288	272
ENTPD1	289	254
DSC1	290	232

KCNQ2	291	152
LRFN1	292	10
IFNGR1	293	299
ZDHHC5	294	233
TSPAN7	295	260
ADGRB1	296	264
HCN1	297	286
DSG1	298	146
CD163	299	298
